# Differential effects of the Akt inhibitor MK-2206 on migration and radiation sensitivity of glioblastoma cells

**DOI:** 10.1186/s12885-019-5517-4

**Published:** 2019-04-03

**Authors:** Cholpon S. Djuzenova, Vanessa Fiedler, Simon Memmel, Astrid Katzer, Dmitri Sisario, Philippa K. Brosch, Alexander Göhrung, Svenja Frister, Heiko Zimmermann, Michael Flentje, Vladimir L. Sukhorukov

**Affiliations:** 1 Department of Radiation Oncology, University Hospital of Würzburg, Josef-Schneider-Strasse 11, 97080 Würzburg, Germany; 2 Department of Biotechnology and Biophysics, University of Würzburg, 97074 Würzburg, Germany; 3 Fraunhofer-Institut für Biomedizinische Technik, Joseph-von-Fraunhofer-Weg 1, 66280 Sulzbach, Germany; 4 Professur für Molekulare und Zelluläre Biotechnologie/Nanotechnologie, Universität des Saarlandes, Campus Saarbrücken, 66123 Saarbrücken, Germany; 5 Marine Sciences, Universidad Católica del Norte, Casa Central, Angamos 0610, Antafogasta/Coquimbo, Chile

**Keywords:** DNA damage, Glioblastoma Multiforme, Histone H2AX, Irradiation, Migration, mTOR, PTEN, p53, Radiation sensitivity, Wound healing

## Abstract

**Background:**

Most tumor cells show aberrantly activated Akt which leads to increased cell survival and resistance to cancer radiotherapy. Therefore, targeting Akt can be a promising strategy for radiosensitization. Here, we explore the impact of the Akt inhibitor MK-2206 alone and in combination with the dual PI3K and mTOR inhibitor PI-103 on the radiation sensitivity of glioblastoma cells. In addition, we examine migration of drug-treated cells.

**Methods:**

Using single-cell tracking and wound healing migration tests, colony-forming assay, Western blotting, flow cytometry and electrorotation we examined the effects of MK-2206 and PI-103 and/or irradiation on the migration, radiation sensitivity, expression of several marker proteins, DNA damage, cell cycle progression and the plasma membrane properties in two glioblastoma (DK-MG and SNB19) cell lines, previously shown to differ markedly in their migratory behavior and response to PI3K/mTOR inhibition.

**Results:**

We found that MK-2206 strongly reduces the migration of DK-MG but only moderately reduces the migration of SNB19 cells. Surprisingly, MK-2206 did not cause radiosensitization, but even increased colony-forming ability after irradiation. Moreover, MK-2206 did not enhance the radiosensitizing effect of PI-103. The results appear to contradict the strong depletion of p-Akt in MK-2206-treated cells. Possible reasons for the radioresistance of MK-2206-treated cells could be unaltered or in case of SNB19 cells even increased levels of p-mTOR and p-S6, as compared to the reduced expression of these proteins in PI-103-treated samples. We also found that MK-2206 did not enhance IR-induced DNA damage, neither did it cause cell cycle distortion, nor apoptosis nor excessive autophagy.

**Conclusions:**

Our study provides proof that MK-2206 can effectively inhibit the expression of Akt in two glioblastoma cell lines. However, due to an aberrant activation of mTOR in response to Akt inhibition in *PTEN* mutated cells, the therapeutic window needs to be carefully defined, or a combination of Akt and mTOR inhibitors should be considered.

**Electronic supplementary material:**

The online version of this article (10.1186/s12885-019-5517-4) contains supplementary material, which is available to authorized users.

## Background

The frequent activation of the PI3K pathway in cancer cells and its crucial role in cell growth and survival has made it a promising target for pharmacologic intervention (for review, *see* [1]). Especially Akt, the most crucial proximal node downstream of the RTK (receptor tyrosinkinase)-PI3K complex, is commonly over-expressed or -activated in all major cancers [2]. Through a number of downstream effectors, such as mTOR, p-4E-BP1 and p-S6K, Akt plays a key role in tumor cell survival and proliferation. Genetic alterations leading to activation of the PI3K/Akt/mTOR pathway are associated with treatment resistance in a variety of solid tumors [3]. Patients whose tumors expressed high levels of p-Akt had decreased survival outcomes and increased metastatic spread after standard chemoradiation [4]. For these reasons, Akt is considered a promising target for cancer therapy and inhibition of Akt alone or in combination with standard cancer chemotherapeutics has been postulated to reduce the apoptotic threshold and preferentially kill cancer cells [2].

The development of Akt inhibitors has been complicated and hampered by the presence of three Akt isozymes (Akt1, Akt2 and Akt3) which differ in function and tissue distribution [5]. Several classes of Akt inhibitors have been developed, including (*i*) lipid-based phosphatidyl-inositol analogues, (*ii*) ATP-competitive inhibitors, and (*iii*) allosteric inhibitors. One member of the allosteric class is MK-2206, an oral, highly selective inhibitor of Akt1, Akt2 and Akt3. MK-2206 binds to the pleckstrin-homology domain of Akt, causing a conformational change that prevents the localization of Akt to the plasma membrane and its subsequent activation [6, 7].

In preclinical models, MK-2206 enhanced the activity of standard chemotherapeutic (carboplatin, gemcitabine, docetaxel, doxorubicin) and molecularly targeted (erlotinib or lapatinib) agents in various lung, breast, ovarian, gastric or hepatocellular carcinoma cell lines [8, 9]. Moreover, combination of MK-2206 and the MEK1/2 inhibitor AZD6244 resulted, through increased apoptosis, in synergistic inhibition of A549 and H157 lung cancer cell growth in vitro and in vivo [10].

Sensitivity to Akt-specific inhibitors is dependent upon activation of the PI3K/Akt/mTOR pathway in tumor cells [11]. Interestingly, catalytic Akt inhibitors (e.g. GDC-0068 and AZD5363) have inhibitory effects in breast tumor cell lines with *Akt* mutations, whereas allosteric inhibitors (e.g. MK-2206) do not exert inhibitory effect on breast, colon and ovarian cancer cells [12, 13]. On the other side, MK-2206, AZD5363 and GDC-0068 have all shown increased activity in cell lines with *PIK3CA* or *PTEN* alterations [14, 15]. This suggests that Akt inhibitors could be indicated for tumors with either *PTEN* loss or *PIK3CA* mutation (for review, *see* [1]). Moreover, MK-2206 alone more potently inhibited cell growth in Ras wild-type cell lines as compared to Ras-mutant NSCLC cell lines [8].

Besides promoting tumor cell proliferation and survival, the Akt/mTOR pathway is recognized as a major pathway regulating autophagy [16], and inhibitors of the Akt/mTOR axis, such as rapamycin analogues, can intensify the autophagic process [17]. Thus, the Akt inhibitor 1 L-6-hydroxymethyl1-chiro-inositol 2(R)-2-O-methyl-3-O-octadecylcarbonate induces authophagic, but not apoptotic cell death, in both radioresistant (U87-MGΔEGFR) and radiosensitive U87-MG glioma cell lines and it enhances sensitivity to radiation [18]. MK-2206 also induces autophagy, as demonstrated by an increase in the 14-kDa form of LC3A/B in hepatocarcinoma cells [9].

In addition to in vitro studies, early-phase clinical trials with allosteric (MK-2206) and catalytic (GDC-0068) Akt inhibitors support the hypothesis that Akt inhibitors can be effective in tumors with PTEN deficiency [1]. Tumor shrinkage has been reported in PTEN-deficient pancreatic cancer cells of patients who received MK-2206 [19].

Beside cytostatic effects, MK-2206 used either alone [20, 21] or in combination with e.g. rapamycin [22] increases radiation sensitivity of various tumor cell lines. Li et al. (2009) showed that Akt inhibition with MK-2206 increased radiation sensitivity of U87-MG cells [20]. Chautard et al. (2010), using a clonogenic survival assay, which was recommended to be named as “percent clone forming test” [23], demonstrated a significant enhancement of radiation sensitivity by an Akt inhibitor IV in human malignant glioma cells [21]. Molecular targeting of Akt by MK-2206 also led to a radiosensitization of lung carcinoma cells which did not respond to the rapamycin (mTORC1 inhibitor) treatment [22]. Moreover, compared to Akt inhibition alone, the dual targeting of Akt1 and mTORC1 markedly enhanced the frequency of residual DNA double-strand breaks (DSBs) by inhibiting the non-homologous end joining repair pathway [22].

We have shown previously that prolonged incubation of several tumor cell lines with the dual PI3K/mTOR-inhibitor PI-103 leads to a re-activation of p-Akt, which in turn might increase tumor cell growth and survival [24]. Given the major roles of Akt in regulating tumor cell survival and resistance to radio- and chemotherapy, we evaluated in this study whether its inhibitor MK-2206 has potential as a radiosensitizing and/or anti-migratory agent in glioblastoma multiforme (GBM) cells. In addition, we examined whether MK-2206 can enhance the radiosensitizing effect of PI-103. We analyzed the impact of MK-2206 alone or in combination with irradiation (IR) and PI-103 on the migration and radiation response of two GBM cell lines differing in *PTEN* and *p53* mutation status. To this end, we analyzed control and drug-treated cells by migration and colony-forming assays, induction and repair of drug- and radiation-induced DNA damage, and cell-cycle phase distribution. We also assessed, by Western blotting, the expression patterns of diverse marker proteins of the PI3K- and MAPK-pathways (p-Akt, p-mTOR, p-4E-BP1, p-S6, p-MEK1/2, p-Erk1/2), as well as DNA repair (ATM, ATR, Rad50 etc.), apoptosis (cleaved PARP, subG_1_-content) and autophagy (LC3B, p62) markers. In addition, plasma membrane properties of control and drug-treated cells were probed by dielectric spectroscopy.

## Methods

### Cell culture

DK-MG (#ACC277) and SNB19 (#ACC325, a subclone of the U-251 MG line according to the provider) human glioblastoma (GBM) cell lines were purchased (on the 7th March 2006 and 04.09.2001, respectively) from DSMZ (Braunschweig, Germany) and authenticated by the supplier. Both GBM cell lines were cultured [24] in the respective complete growth medium (CGM) containing 10% fetal bovine serum according to provider’s prescription. Cells were used at low (< 15) passages after thawing and were regularly authenticated on the basis of morphology, growth curve analysis, expression of PTEN and p53; and were regularly examined during the study for Mycoplasma (MycoAlert; Lonza, Rockland, ME, USA).

### Inhibitor treatment

Both inhibitors were purchased from Selleckchem (Absource Diagnostics GmbH, Munich, Germany). The substances were freshly diluted from frozen (− 80 °C) aliquots dissolved in DMSO. MK-2206 (5 μM, [14]) and PI-103 (2 μM, [24]) were added alone or in combination 3 h (short-term treatment) prior to exposure to ionizing radiation (IR) and remained in CGM up to 24 h (long-term treatment) after IR. Control cells were treated in parallel with respective amounts of DMSO.

### Antibodies

The primary and secondary antibodies used in this paper are indicated in the Additional file 1: Supplemental Materials.

### Irradiation treatment

Irradiation of the samples was performed at room temperature using a 6 MV Siemens linear accelerator (Siemens, Concord, CA) at a dose rate of 2 Gy min^− 1^. After exposure to IR, cells were further cultured in CGM for the indicated time until processing [24].

### Phase-contrast microscopy of living cells

A Nikon BioStation IM-Q (Nikon, Melville, NY), which includes a cell incubator (37 °C, 5% CO_2_), a motorized inverted phase contrast microscope and a high-sensitivity CCD camera, was used to image live GBM cells over time [25]. Prior to single-cell tracking experiments, cells (about 10^4^ in 2 ml medium) were plated into a Petri dish (diameter 35 mm). Time-lapse images of several fields (706 × 530 μm) of view were acquired in each experiment every 10 min over an 18-h period, using a 10x phase contrast objective.

### Individual cell tracking and analysis of migration

Individual cell tracking was performed using the Time Lapse Analyzer (TLA; University of Ulm, Germany) software, tailored for a tracking procedure with automatic cell identification of unstained cells in phase contrast microscopy videos. Image analysis, including pre-processing and segmentation for the separation of the cells from the background, and track generation were performed essentially as described elsewhere [25]. Using the acquired track information, cell migration speed and directionality of migration were calculated for each individual cell. Motility speed was calculated as the total distance (TD) a cell travelled divided by the total movement time. Migration directionality was assessed as the net distance (ND), which is the shortest way from the starting position to the end of migration path, where 1.0 corresponds to migration in a straight line, and 0.0 to migration with an identical start and endpoint (circular or no movement).

### Wound healing (scratch) test

Wound closure in the control or drug-treated cell samples, non-irradiated or with IR treatment, was examined up to 18 h after scratching as described elsewhere [25]. A wound or scratch was made with a sterile micropipette tip through confluent cell monolayers plated on a glass dish. Video images were acquired using a Biostation IM-Q (Nikon, Melville, NY). Wound closure rates were quantitated with Image J (Wayne Rasband, National Institutes of Health, Bethesda, MD).

### Colony-forming test

To assess the effect of IR, a standard colony-forming assay was performed as previously described [24]. Control and drug-treated cells were irradiated with a single dose (0, 2, 3, 5, 7 or 8 Gy) of irradiation and seeded 24 h post-IR in Petri dishes for further cultivation. After about 10–12 days, fixation (methanol/acetic acid) and staining with a solution of 0.6% *w*/*v* crystal violet was performed [24]. Only colonies containing more than 50 cells were counted. The mean colony-forming data for each tested cell line were fitted to the linear-quadratic (LQ) model (Eq. 1):

1 $$ CFF=\exp \left(-\alpha X-\beta {X}^2\right) $$

where *CFF* is the colony-forming fraction, *X* is the irradiation dose, and *α* and *β* are the linear and quadratic terms of the fitting function. Both the LQ and the LQ-L (linear-quadratic linear) are state-of-the-art methods for the analysis of colony-forming ability [26]. Usually a very good agreement between the observed experimental data and the estimated regression curves is obtained.

### DNA damage and cell-cycle measurements detected by flow cytometry

Control and drug-treated subconfluent cell cultures were irradiated with a single radiation dose of 8 Gy and analyzed by flow cytometry 30 min and 24 h post-IR. A single dose of 8 Gy used in the study is far higher than the single daily fraction during radiotherapy of cancer patients, which ranges between 1.8 and 2 Gy resulting in a total dose of 50–60 Gy after 4–5 weeks. However, in order to cause detectable cell cycle disturbances and/or to enable the detection of γH2AX by flow cytometry or apoptosis much higher irradiation doses in the range of 5–12 Gy are required in radiobiological experiments (e.g. [27–29]). In our experiments, single irradiation with the clinically relevant dose of 2 Gy did not induce any cell cycle alterations in SNB19 cells detectable by flow cytometry, both 30 min and 24 h after IR, as compared to non-irradiated controls (Additional file 2: Table S1). In sharp contrast, 24 h after irradiation with 8 Gy the cells exhibited a marked G_2_-arrest. Likewise, irradiation with 8 Gy had a more pronounced effect on the γH2AX content (both initially induced and residual) detected by flow cytometry as compared to cells irradiated with 2 Gy in glioblastoma SNB19 cells (Additional file 2: Table S2). At indicated times after IR, cells were detached by trypsinization, washed in PBS, fixed and stained for DNA damage marker histone γH2AX as previously described [24]. In addition, the samples were stained with propidium iodide (PI, Sigma P-4170, 10 μg ml^− 1^) in the presence of ribonuclease A (Sigma R-5250, 25 μg ml^− 1^) to avoid RNA staining. Red (PI-DNA) and green (histone γH2AX) fluorescence was acquired in linear or logarithmic mode, respectively, using a flow cytometer FACSCantoII (Becton Dickinson, San Jose, CA). The samples include both floating and trypsinized cells. The output data were presented as one-dimensional histograms, i.e. the distributions of PI-DNA or histone γH2AX signals within cell samples and analyzed using ModFit LT (Verity Software House, Topsham, ME) and Flowing Software obtained from P. Terho (Turku Centre for Biotechnology, Turku, Finland). In addition, the sub-G_1_ fraction was evaluated to assess the late-stage apoptosis.

### Western blotting

The expression of marker proteins was tested using western blotting as previously described [24]. Cellular lysates were prepared 30 min and 24 h post-IR according to standard procedures. Forty μg of protein per lane were separated using 4–12% SDS-polyacrylamide pre-cast gels (Invitrogen, Karlsruhe, Germany) and transferred to nitrocellulose membranes according to the manufacturer’s prescriptions. The levels of protein expression were quantified using Image J (Wayne Rasband, National Institutes of Health, Bethesda, MD) and normalized to β-actin intensity. Experiments were repeated at least three times, unless otherwise noted.

### Electrorotation and derivation of membrane parameters

Measurements of the electric field frequency *f*_c1_ that produced the fastest anti-field rotation of cells were carried out in isotonic (300 mOsm) inositol medium with the contra-rotating fields (CRF) method as described previously [30]. The theory of electrorotation predicts the following relationship between the characteristic frequency of anti-field electrorotation *f*_c1_, the cell radius *a*, the membrane capacitance *C*_m_ [μF/cm^2^] and conductance *G*_m_ [mS/cm^2^] per unit area:

2 $$ {f}_{\mathrm{c}1}\cdot a=\frac{\sigma_{\mathrm{e}}}{\uppi \cdot {C}_m}+\frac{\left\langle a\right\rangle \cdot {G}_{\mathrm{m}}}{2\uppi \cdot {C}_{\mathrm{m}}} $$

where *a* and < *a* > are, respectively, individual and mean cell radii, *σ*_e_ [μS/cm] is the conductivity of suspending medium, which is much lower than the intracellular conductivity (i.e. *σ*_e_ < < *σ*_i_). The membrane quantities *C*_m_ and *G*_m_ can be derived by fitting Eq. 2 to the (*f*_c1_·*a*) data plotted against *σ*_e_ [30].

From the *C*_m_ and cell radius values, the whole-cell capacitance *C*_C_ [pF] was also calculated as:

3 $$ {C}_C=4\uppi \cdot {\mathrm{a}}^2\cdot {C}_{\mathrm{m}} $$

Unlike the *C*_m_ value, which represents the area-specific membrane capacitance, the parameter *C*_C_ accounts for the total electrically accessible cell membrane, including both smooth and folded membrane regions.

### Statistical analysis

Data are presented as mean ± SE, unless otherwise stated. Student’s *t*-test was performed when statistical comparisons were made between two sets of data. Difference with *p*-values of <0.05 were considered significant.

## Results

### Effects of Akt-, PI3K-, and mTOR-inhibition on the migration of GBM cells

**Fig. 1:**
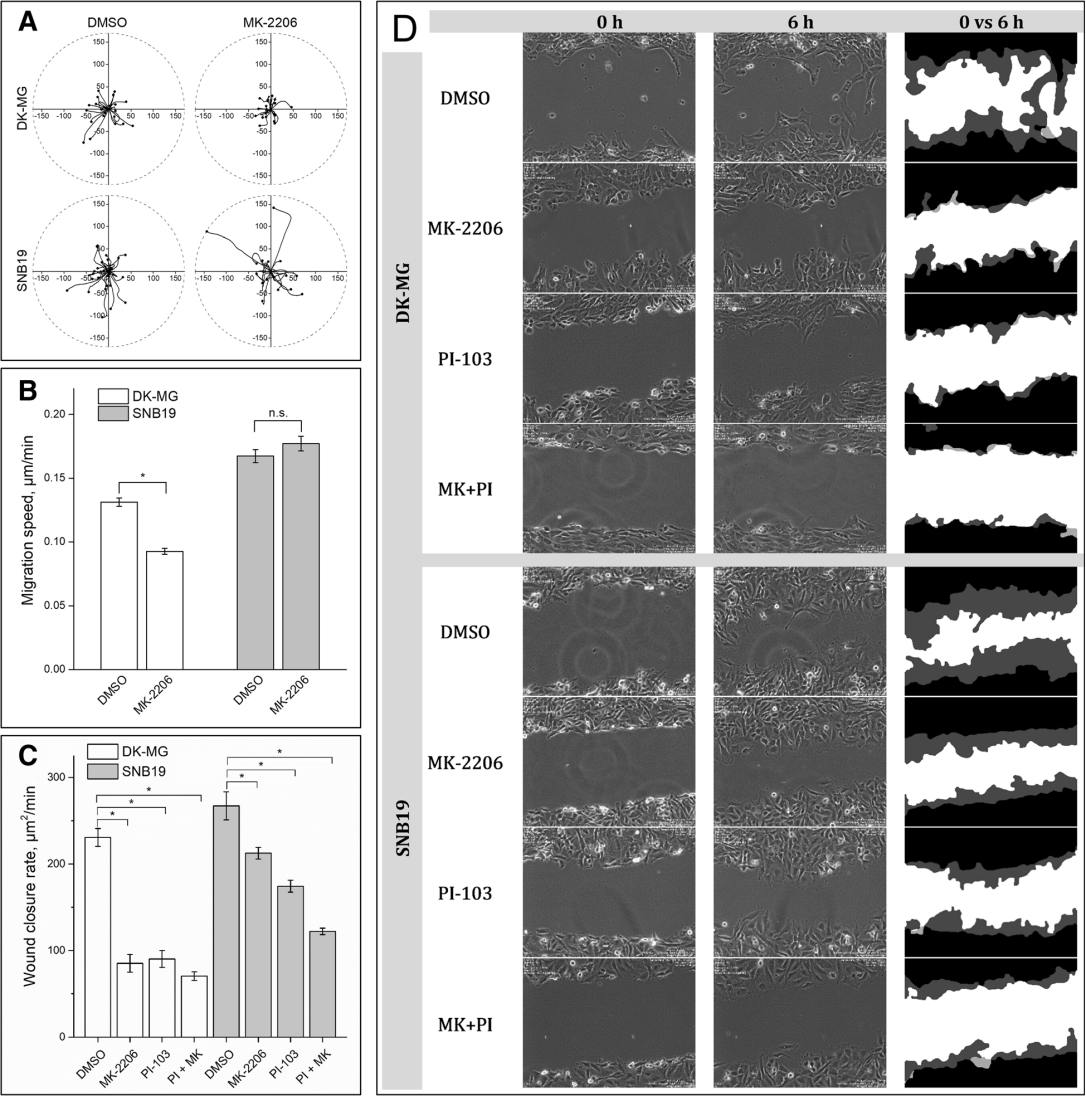
Impact of Akt and/or PI3K/mTOR inhibition on the migratory behavior of DK-MG and SNB19 cells assessed via single-cell tracking (**a**, **b**) and wound healing (**c**, **d**) tests. Part **A** illustrates migration paths of 20–30 representative cells during 5 h, for each treatment. For each cell, its initial position was set to the origin of coordinate system. Black circles denote the final cell locations at *time* = 5 h. The corresponding time lapse videos are given in Additional file 3: movie S1. Part **b** summarizes the mean (± SE) migration speed values (i.e. *total* distance, TD divided by time) from at least 400 cells per treatment. The bar graphs in **C** depict the impact of inhibitor treatment on the wound closure rate in DK-MG and SNB19 cell monolayers, expressed in unit area per min [μm^2^/min]. Each bar represents the mean ± SE of at least three independent experiments, each performed four times. Phase contrast images of GBM cells shown in part **d** were acquired at 0 and 6 h after the wound was made. White color in the right-side column of **d** represents the cell-devoid area, black and grey colors show areas covered with cells at *time* = 0 and 6 h, respectively. “*” stands for *p* < 0.05. “n.s.” means “not significant”

We recently reported that the migration activity of SNB19 cells is much less responsive to PI3K/mTOR inhibition than that of DK-MG cells [25]. In the present study, we tested whether Akt inhibition can reduce the motility of SNB19 cells. First, we examined the migration of individual DK-MG and SNB19 cells (Fig. 1 and Additional file 3: Movie S1) by time-lapse video-microscopy [25].

As evident from the motility diagrams shown in Fig. 1a and Additional file 3: Movie S1, which are statistically summarized in Fig. 1b, treatment with MK-2206 decreased the migration speed of DK-MG cells by ~ 30% with respect to controls cells. In contrast, MK-2206 did not affect the migration activity of SNB19 cells (Fig. 1b). Data on the effects of PI-103 have been previously reported [25]. The effects of both inhibitors on the migration of irradiated cells were similar to their effects on non-irradiated samples from both cell lines (data not shown).

Additionally to individual cell tracking, we analyzed cell movement by wound healing (scratch) assay. Representative data of the wound healing tests on both cell lines shown in Fig. 1d are statistically summarized in Fig. 1c. Similarly to the results of the single-cell tracking assay, drug-treated DK-MG cells closed the wounded area much slower (by ~ 65–70%) than control cell samples (Fig. 1c, d).

A further interesting finding of the wound healing test was that the Akt-inhibitor was less effective in SNB19 cells (Fig. 1c, d). Thus, treatment with MK-2206 alone only moderately (~ 20%) affected the wound closure rate of SNB19 cells. Besides this, compared to drug-free controls, treatment of SNB19 cells with PI-103 alone and with both drugs caused, respectively, an ~ 35 and ~ 45% decrease in the wound healing rate compared with the control cells (Fig. 1c), which is much less than the almost 70% inhibition observed in drug-treated DK-MG cells. Qualitatively similar data were obtained on irradiated cells (data not shown).

### Effect of MK-2206 alone and in combination with PI-103 on colony-forming ability after irradiation

**Fig. 2:**
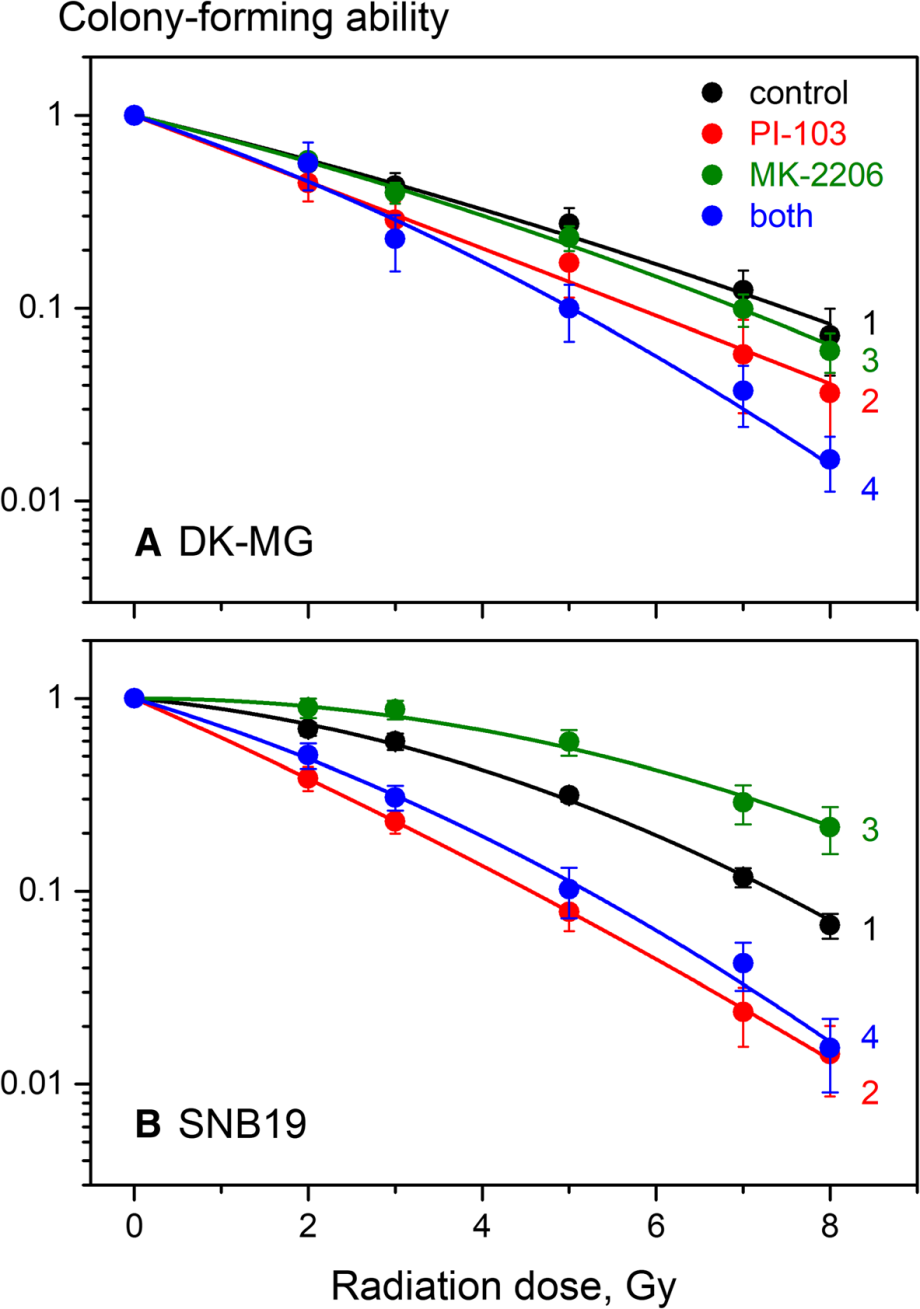
Colony-forming ability of irradiated DK-MG (**a**) and SNB19 (**b**) cells pre-treated for 3 h prior to IR with MK-2206, PI-103 or both. Cells were seeded for the colony-forming test 24 h after IR. Ten to twelve days later, colonies containing at least 50 cells were counted as survivors. Data derived from at least five independent experiments for each cell line (each performed four times) were pooled together and fitted to a linear-quadratic equation (Eq. 1). The SE values are indicated by error bars. “*” means *p* < 0.05. “n.s.” means “not significant”

We further analyzed whether the Akt inhibition alone or in combination affected the radiation sensitivity of tumor cells. Figure 2 shows the normalized colony-forming curves of untreated and drug-treated cells plotted against the radiation dose, along with the best fit curves of the LQ model (Eq. 1) to the survival data. The plating efficiencies (PE) of control cell samples, as well as the values obtained with the LQ model, including the colony-forming fraction at 2 Gy (CF2), the radiation exposure dose required to reduce colony-forming ability by 10% (D_10_) and the growth inhibition factor (IF_10_) are summarized in Additional file 2: Table S3.

As seen in Fig. 2 and in accordance with our previous results [24], PI-103 alone acts as a potent radiosensitizer in both cell lines (Fig. 2, curves 2 in parts A and B). In contrast, MK-2206 given alone had little - if any - effect on the radiation sensitivity of DK-MG cells (curve 3, Fig. 2a) and, unexpectedly, it significantly *increased* the radiation resistance of SNB19 cells (curve 3, Fig. 2b). Upon combined drug treatment, MK-2206 only slightly enhanced the radiosensitizing effect of PI-103 in DK-MG cells (Fig. 2a, curve 4), without affecting the PI-103-mediated radiosensitization in SNB19 cells (Fig. 2b, curve 4).

### Effects of MK-2206, PI-103 and/or irradiation on cell cycle

**Fig. 3:**
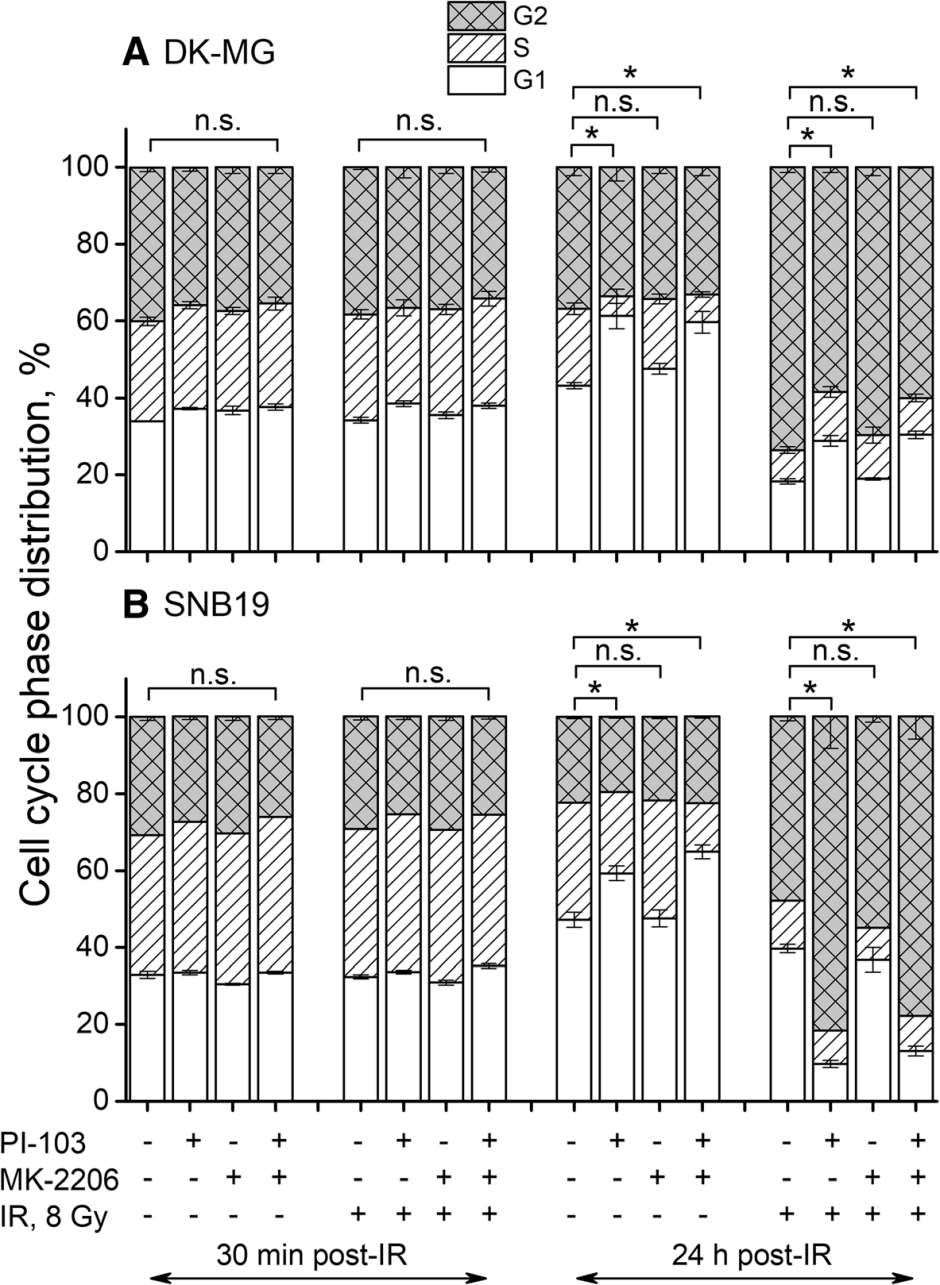
Cell cycle-phase distribution in DK-MG (**a**) and SNB19 (**b**) tumor cells treated for 3 h with PI-103, MK-2206 either alone or in combination and irradiated with 8 Gy. Thirty minutes and 24 h after IR cells were fixed, permeabilized, stained with propidium iodide, and analyzed for DNA content by flow cytometry. Data are presented as means (± SE) of at least three independent experiments

To find the molecular basis and to identify the mechanisms underlying the absence of the radiosensitizing effect of the Akt inhibitor MK-2206 in DK-MG cells (Fig. 2a) or the radioresistance in respective SNB19 cells (Fig. 2b) the attempts were focused on the inhibitor’s possible impact on cell cycle progression. The data for both cell lines are summarized in Fig. 3. The large fractions of cells in the S- and G_2_/M-phases (> 70%) in control non-irradiated probes (Fig. 3) show that the cells were growing exponentially at the start of experiments. As expected, 30 min post-IR, non-irradiated and irradiated cells (within the respective cell line) exhibited similar cell cycle phase distribution (Fig. 3). However, as shown previously [24], prolonged incubation with PI-103 caused an increase of G_1_ phase cells from about 40% to ~ 60%. In contrast, upon a long-term incubation with MK-2206, the cell cycle phase distributions in both cell lines were nearly the same as in respective non-treated controls (Fig. 3). Combined prolonged drug treatment caused a strong G_2_/M arrest in irradiated samples of both cell lines similar to that observed after treatment with PI-103 alone and IR.

The cell cycle impairment caused by PI-103 alone and in combination shown above (Fig. 3) led us to determine the expression levels of several cell-cycle regulating factors such as cyclin dependent kinases (Cdk1, Cdk4), and pRb by Western blotting. As shown in the Additional file 4: Figure S1, the expressions of Cdk1 and pRb decreased although to a different extent in both GBM lines after prolonged exposure to PI-103 alone or in combination, especially in non-irradiated cells. In contrast, the levels of cell cycle-related proteins were mostly unchanged in non-irradiated cells treated with MK-2206 alone.

To sum up, long-term incubation with PI-103 caused a significant G_1_ arrest in both cell lines, but combined with IR it produced a strong G_2_/M arrest. In contrast, treatment with MK-2206 alone had no influence on the cell cycle. In both cell lines, combined treatment with both drugs and IR caused a profound G_2_/M arrest 24 h post-IR, similar to that induced by PI-103 alone and IR.

### Effects of MK-2206 and PI-103 and/or radiation on late-stage apoptosis and autophagy

**Fig. 4:**
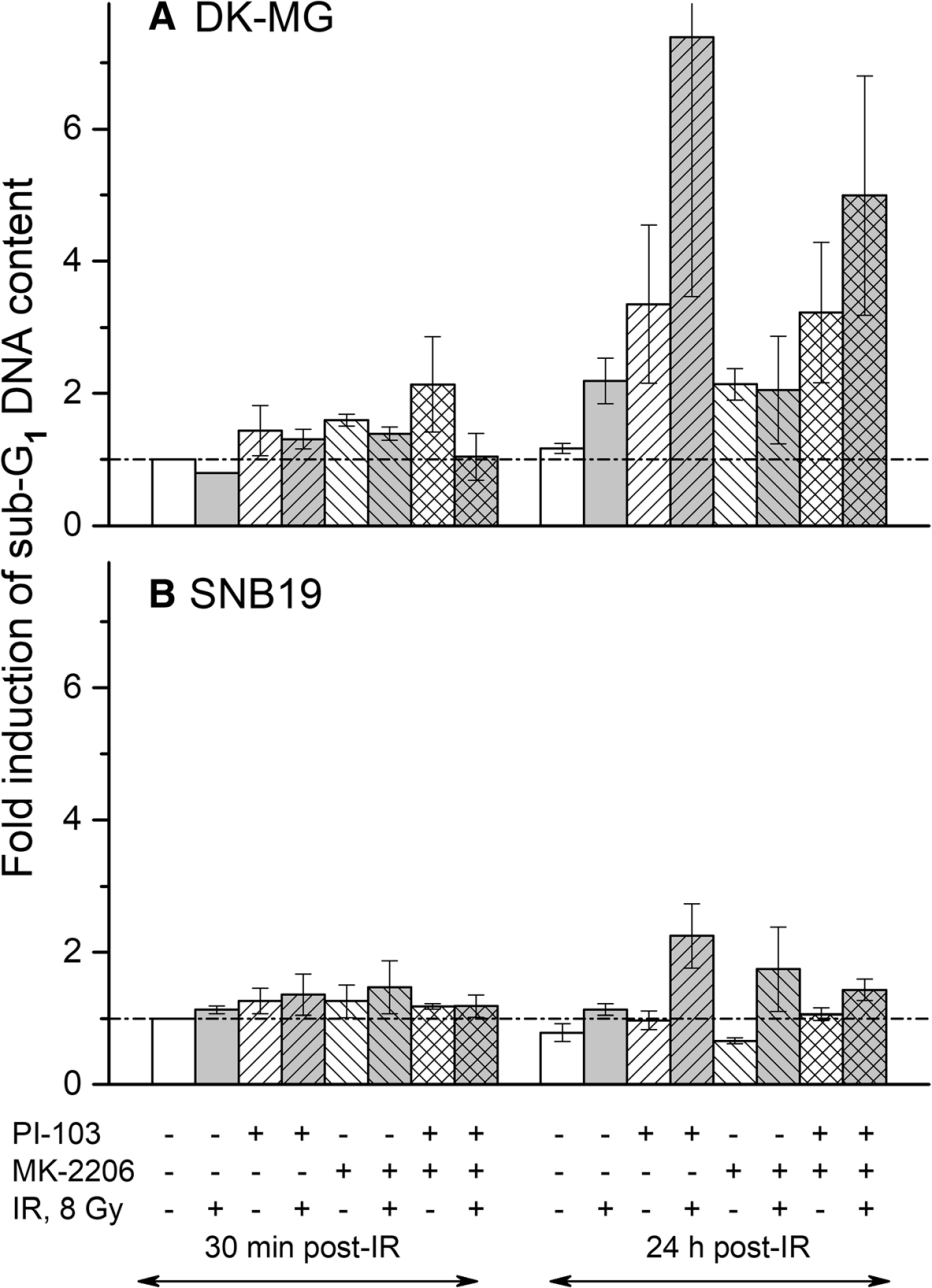
Flow-cytometric analyses of the sub-G_1_ DNA content distribution in DK-MG and SNB19 cells subjected to drug-IR treatments were performed 30 min or 24 h after IR. The cells were detached with trypsin, treated with saponin and RNAse, stained with propidium iodide and then analyzed for red fluorescence by flow cytometry. The samples include both floating and trypsinized cells. Changes in the sub-G_1_ fraction are depicted as folds of increase (means ± SE), normalized to respective non-irradiated drug-free controls (30 min post-IR). The sub-G_1_ fraction includes hypodiploid nuclei and debris, computed from the flow cytograms with the Flowing Software for *n* = 3 independent experiments

To further dissect the mechanisms underlying the radiation survival of GBM cells after single Akt- and combined PI3K/Akt/mTOR-inhibition illustrated in Fig. 2, we also analyzed cleaved PARP, a well-known marker of apoptosis. As seen in Additional file 4: Figure S2, DK-MG cells treated with PI-103 alone or in combination with MK-2206 exhibited negligible levels of cleaved PARP, independent of IR, whereas no any cleaved PARP was detected in SNB19 samples. Likewise, the Akt-inhibitor did not induce any measurable amounts of cleaved PARP. In addition, the apoptosis rate was evaluated by the sub-G_1_ fraction. As seen in Fig. 4, inhibitor-treated and irradiated DK-MG samples contained much larger fractions of apoptotic cells and debris than corresponding SNB19 cell samples, especially 24 h after IR.

Because the Akt/mTOR pathway is recognized as a major pathway regulating autophagy [16], we also studied the role of autophagy in the development of radiation resistance in MK-2206-treated cells, especially in case of the SNB19 cell line. To this end, we detected the autophagosomal membrane-bound LC3B protein along with the expression of the p62/sequestome protein, a pleiotropic protein that is consumed during autophagy [31]. As seen in the Additional file 4: Figure S3, IR increased the expression of LC3B-I and LC3B-II proteins in both cell lines. This result is corroborated by earlier findings that radiation enhances autophagy in tumor cells (for review, *see* [32]). We also found that the dual PI3K- and mTOR-inhibitor PI-103 alone and especially in combination with MK-2206 strongly induced autophagy, as evident from the increased levels of LC3B-II protein (Additional file 4: Figure S3). However, the ratio between LC3B-II and LC3B-I was lower in irradiated cells compared to non-irradiated. Furthermore, the enhanced autophagy in drug-treated samples, detected by LC3B-II expression, was also corroborated by the strong depletion of p62 protein, another marker of autophagy (Additional file 4: Figure S3). These findings agree well with the results of Fan et al. (2017) who found that dual inhibition of PI3K and mTOR promotes survival of glioma cells by inducing cytoprotective autophagy [33]. Regarding MK-2206, the extent of drug-induced autophagy measured by LC3B-II expression was lower than that induced by PI-103. Likewise, when assessed by p62 expression, MK-2206 alone induced autophagy to a much lesser extent than PI-103. The highest extent of autophagy was observed in samples treated with both inhibitors simultaneously. Interestingly, the background expression of p62 in DK-MG cell line was much lower than in SNB19 cells. The difference may reside in the wild type status of p53 in DK-MG cells because functional p53 is known to inhibit autophagy [34].

### Effects of MK-2206 and PI-103 and/or radiation on the expression of marker proteins

**Fig. 5:**
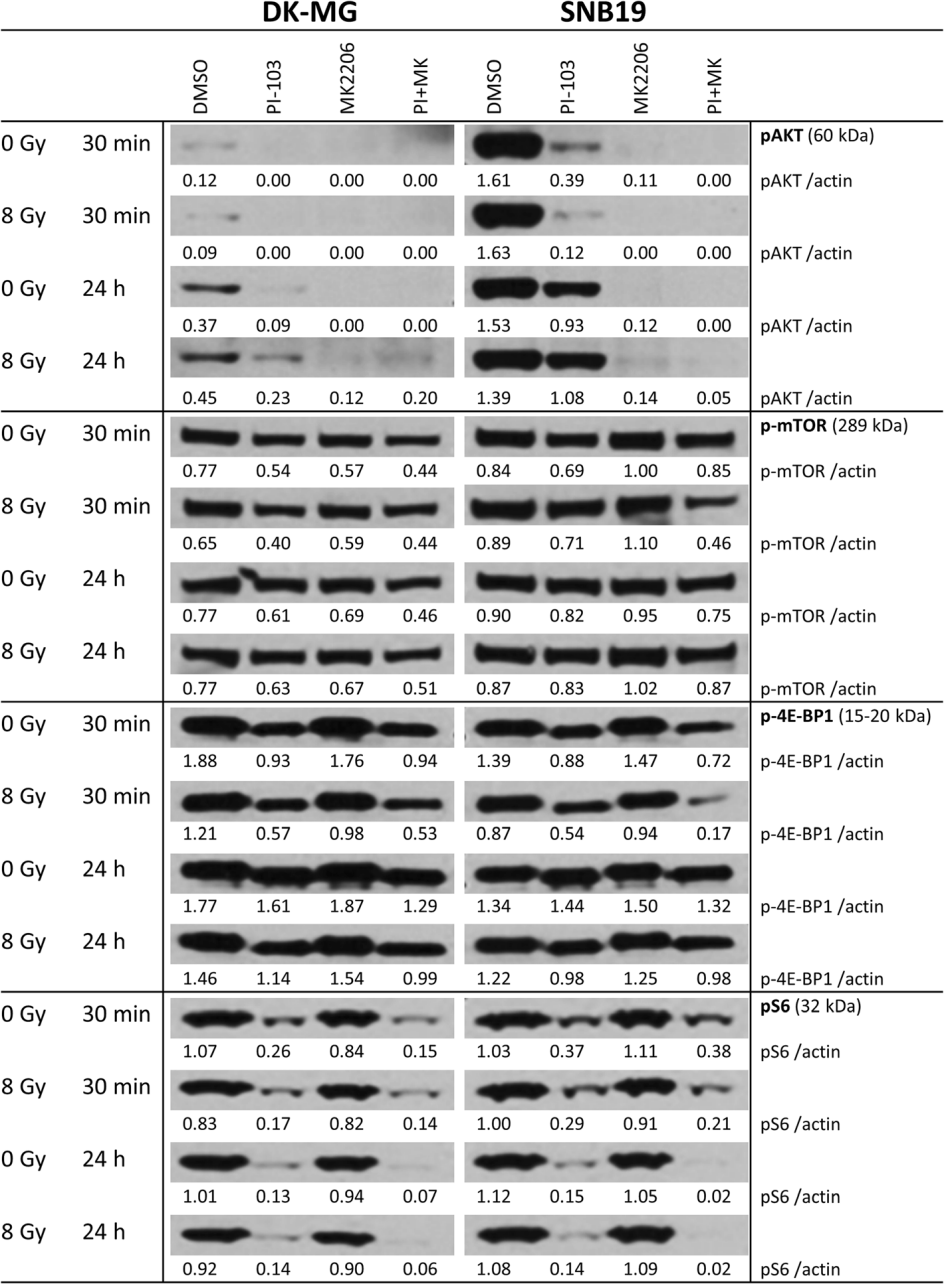
Expression patterns of various marker proteins of PI3K-pathway in DK-MG and SNB19 cells treated with DMSO (control) or the indicated compounds for 3 h before IR with 8 Gy and detected 30 min and 24 h thereafter. Each protein band was normalized to the intensity of β-actin used as loading control. The protein/β-actin ratios are indicated by the numbers. The Western blot experiments were repeated independently at least three times

To further investigated the molecular basis for the observed effects in MK-2206-treated tumor cells with or without addition of PI-103, or IR exposure (Figs. 1, 2), we studied the expression of two groups of proteins. The first group (Fig. 5 and Additional file 4: Figure S4) includes several marker proteins of the PI3K-pathway, i.e. p-Akt and mTOR, along with p-4E-BP1 and p-S6. The second group includes two proteins of the MAPK-pathway, i.e. MEK1/2 and Erk1/2 (Additional file 4: Figure S5). Figure 5 shows exemplarily Western blot data of control and drug-treated cells probed for p-Akt, p-mTOR, p-S6 and p-4E-BP1 proteins 30 min and 24 h after IR with 8 Gy. Samples shown on the left- and right-hand sides (LHS, RHS) of Fig. 5 were obtained from DK-MG and SNB19 cells, respectively.

Complete loss of *PTEN* in *PTEN-*mutated SNB19 cells, commonly leads to an over-activation of the PI3K pathway. As seen in Fig. 5 (RHS column), the expression of p-Akt in SNB19 cells was much higher than in DK-MG cells containing wild type *PTEN*. Addition of MK-2206 or PI-103 either alone or in combination strongly reduced the p-Akt level after a 3-h drug treatment, with and without IR. However, in accordance with our previous findings [24], prolonged treatment with PI-103 caused reactivation of the Akt function in both cell lines. Thus, p-Akt expression (1.1 a.u.) in SNB19 cells almost recovered to the background level of 1.4 a.u. (Fig. 5, RHS). In contrast, both cell lines remained depleted of p-Akt after prolonged treatment with MK-2206 alone or in combination with PI-103, independent of IR exposure.

In addition to p-Akt, we analyzed the expression of p-mTOR and its downstreams, ribosomal S6 and translational repressor 4E-BP1 proteins, which are known to influence cell-cycle progression and cell growth [35, 36]. The expression of p-mTOR *decreased* after short incubation with PI-103 alone or in combination with MK-2206 (Fig. 5) in both cell lines. Upon long-term exposure to both drugs, p-mTOR expression almost returned to the control level in SNB19 cells, but not in DK-MG cells. As a result of mTOR inhibition by PI-103, both p-4E-BP1 and p-S6 were also strongly suppressed 30 min or even depleted (p-S6) 24 h post-IR (Fig. 5) in cells treated with PI-103 alone and especially in combination with MK-2206.

Contrary to the *inhibition* of p-mTOR by PI-103, MK-2206 moderately *increased* the p-mTOR level in SNB19 cells (but not in DK-MG cells) 30 min post-IR (1.0–1.1 a.u.), as compared to control (0.84–0.89 a.u.) and especially to PI-103-treated cells (0.69–0.71 a.u.; Fig. 5). As a result, p-4E-BP1 and p-S6 proteins were also increased after addition of MK-2206 alone to SNB19 cells, whereas they were strongly reduced or even vanished after prolonged PI-103 treatment. At the same time, in DK-MG cells the expressions of p-mTOR and p-4E-BP1 remained mostly unchanged (30 min and 24 h post-IR) in the samples treated with MK-2206 alone or in combination with PI-103. The corresponding bands of β-actin are shown in Additional file 4: Figure S4.

Because of the mutual dependence of the PI3K and MAPK pathways [37], we also analyzed two kinases of the MAPK signaling pathway, p-MEK1/2 and p-Erk1/2 (Additional file 4: Figure S5). The MAPK pathway, which is frequently mutated in cancer cells [38, 39], transmits signals from surface receptors to stimulate cell survival, proliferation and migration [40]. We found that a short incubation with MK-2206 alone or in combination with PI-103 slightly increased the expressions of p-MEK1/2 and p-Erk1/2 (Additional file 4: Figure S5). However, after prolonged incubation with MK-2206 alone or in combination with PI-103 the effect was less evident.

Next we studied Rheb protein, which was shown to activate mTORC1 in vitro [41]. As seen in Additional file 4: Figure S6, after a short incubation with MK-2206 the Rheb expression was increased in SNB19 but not in DK-MG cells. At 24 h post-IR the expression of Rheb was higher in all drug-treated and irradiated probes of SNB19 cells compared to non-treated controls. At the same time, the expression of Rheb in DK-MG cells remained mostly unchanged by drug application.

### Effects of MK-2206 and PI-103 and/or radiation on DNA damage

**Fig. 6:**
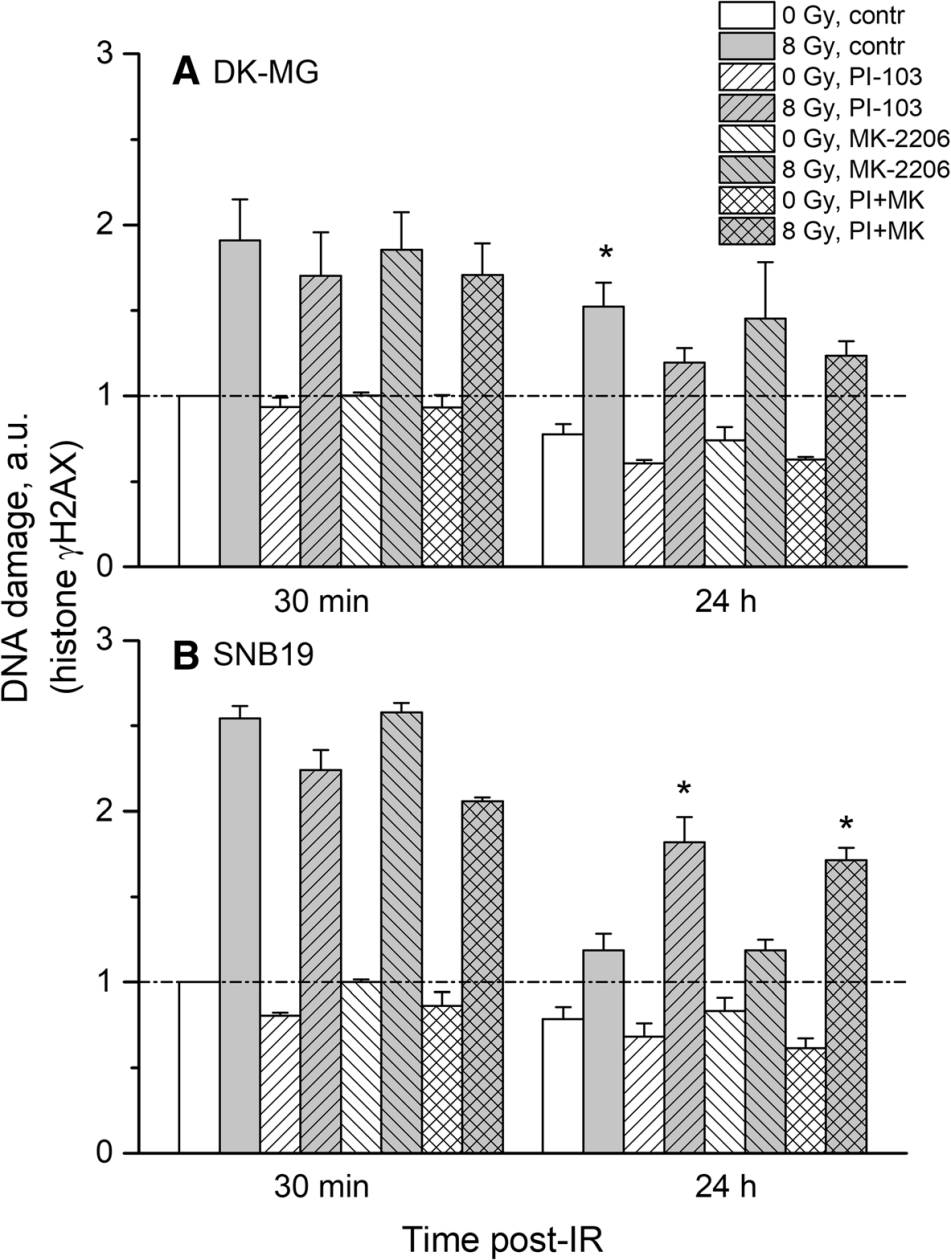
DNA damage in control and drug-treated and/or irradiated DK-MG (**a**) and SNB19 (**b**) cells assessed by histone γH2AX 30 min and 24 h post-IR with 8 Gy and quantified by flow cytometry. The bar graphs are the means (± SE) of at least 3 independent experiments. The data of each cell line are normalized to the initial γH2AX content (at 30 min post-IR) detected in drug-free non-irradiated controls. “a.u.” means arbitrary units, “*” means *p* < 0.05

To elucidate the cause for the radioprotective effect of the Akt inhibitor MK-2206 in colony-forming tests, especially in SNB19 cells (Fig. 2), we evaluated DNA damage in control and drug-treated cells after IR. The results are statistically summarized in Fig. 6 as the mean (± SE) values of the amount of DNA damage detected 30 min and 24 h after IR in all cell probes. As seen in Fig. 6a, irradiated drug-free DK-MG cells showed increased residual damage 24 h post-IR. However, no differences in the induction and repair of DNA damage were found between irradiated DK-MG cells independent of drug pretreatment. On the contrary, in irradiated SNB19 cells (Fig. 6b) the residual (24 h post-IR) damage to DNA after addition of PI-103 alone or in combination with MK-2206 was much higher than in drug-free irradiated cells. Interestingly, MK-2206 alone did not influence the degree of induced or residual DNA damage in both cell lines.

**Fig. 7:**
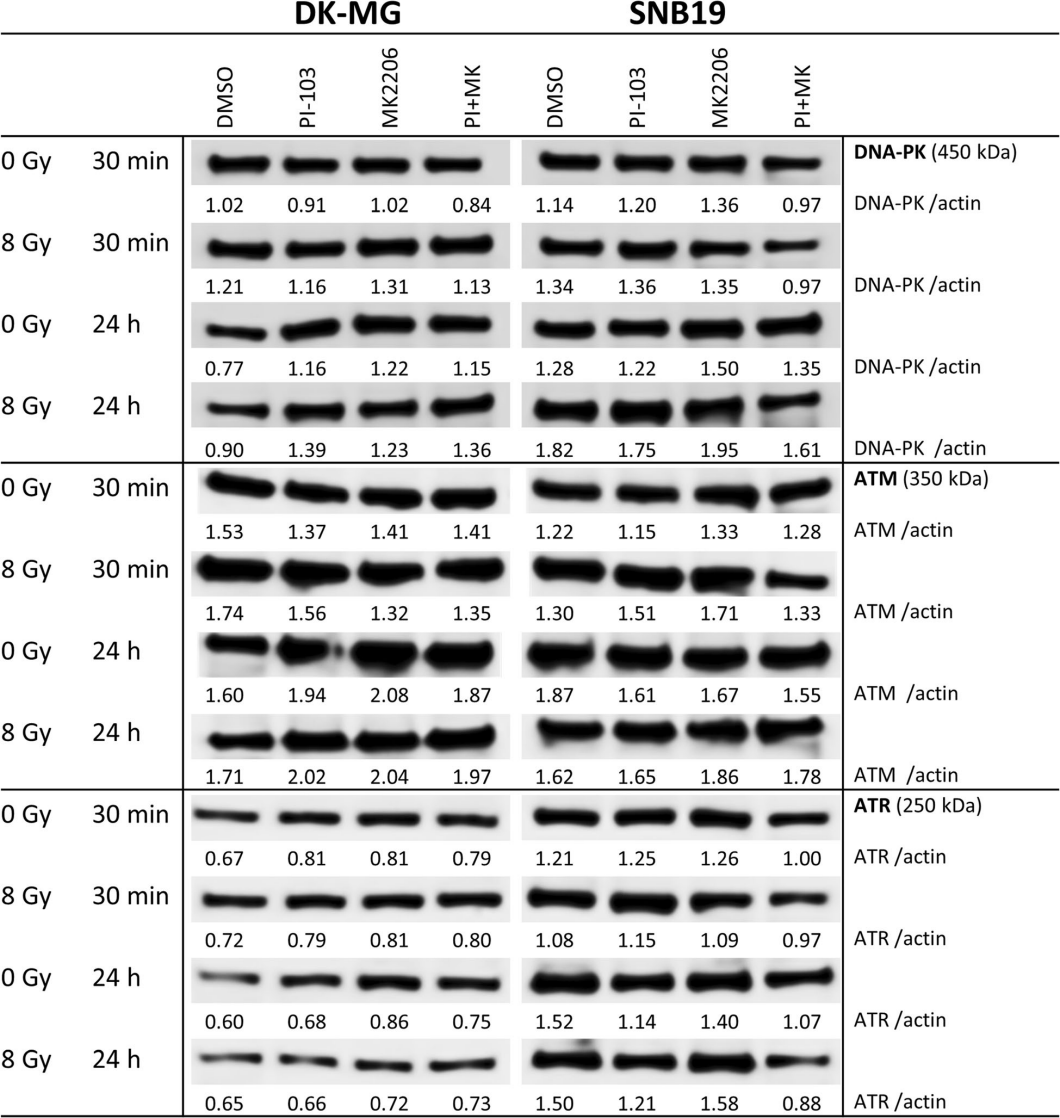
Representative Western blots of several DNA repair proteins in two tumor cell lines subjected to 3-h pretreatment with both tested drugs before IR. Cell lysates were prepared 30 min and 24 h after irradiation with 8 Gy. For details, *see* legend to Fig. 5

Driven by the finding that both inhibitors added alone differently affect the repair of DNA damage in irradiated SNB19 cells (Fig. 6b), without influencing the repair process in DK-MG cells (Fig. 6a), we analyzed the expression of several DNA repair proteins. Figure 7 shows representative Western blot detections of several DNA repair proteins in both cell lines treated with drugs and IR. Thirty minutes after IR, the expression levels of DNA-PK, ATM and ATR proteins were very similar in control and drug-treated samples of DK-MG cells (Fig. 7, LHS). However, 24 h post-IR the expression of DNA-PK and ATM proteins was higher in drug-treated than in drug-free DK-MG cell samples. On the contrary, drug treatment did not cause any changes in the expression of DNA-PK and ATM in SNB19 cells (Fig. 7, RHS). The expression of ATR was mostly unchanged throughout the experiment in both cell lines, except within irradiated SNB19 cells treated for more than 24 h with PI-103 alone or in a combination.

Furthermore, we studied the expression of DNA repair proteins Rad50, Rad51, Ku70 and Ku80 (Additional file 4: Figure S7). We found no differences in the expression of Rad50, Ku70 and Ku80 in both cell lines throughout the whole study. In contrast, the expression of Rad51 was strongly reduced in non-irradiated samples of both cell lines treated with PI-103 alone or in combination with MK-2206 (Additional file 4: Figure S7). Given that Rad51 operates mostly in the G_2_ phase of the cell cycle [42], the decrease in Rad51 can be explained by the G_1_-arrest after addition of PI-103. However, despite the strong reduction of Rad51 in both cell lines, DK-MG cells showed normal repair of DNA damage, whereas SNB19 cells showed protracted DNA repair. This means that the impaired DNA repair capacity revealed by the high residual histone γ-H2AX levels in SNB19 cells treated with PI-103 alone or in combination with MK-2206 (Fig. 6b) cannot be explained by the reduction of Rad51 (Additional file 4: Figure S7, RHS). The corresponding blots for β-actin expression are shown in Additional file 4: Figure S8.

### Effects of MK-2206 and PI-103 on the plasma membrane properties probed by electrorotation (ROT)

In a series of studies, we have shown recently that the two glioblastoma cell lines (DK-MG and SNB19) studied here are very different not only in their invasiveness and migratory behavior [25, 43], but also in their morphological appearance including membrane surface area and folding [44], analyzed by electron microscopy and electrorotation. Although derived from the same tumor entity, the two cell lines differ markedly in the mutational status of the most prominent tumor suppressors *PTEN* and *p53* [43, 44]. Both genes are wild type in DK-MG cells, whereas in the SNB19 cell line both genes are mutated, which leads to a marked difference between the two cell lines regarding their phospholipid and membrane synthesis (for detail, *see* Fig. 7 in [44]). Furthermore, the two cell lines differ greatly from each other in their response to the pharmacological inhibition of PI3K and mTOR in terms of migration [25]. Taken together, the above mentioned findings prompted us to analyze in this study the effects of the PI3K-Akt-mTOR inhibition not only on GBM cell migration but also on the plasma membrane morphology, which is closely related to cellular motility and invasion [43].

As shown above (Fig. 5), a 3-h incubation with MK-2206 had different effects on the mTOR expression in tested cell lines, i.e. an up-regulation in SNB19 cells and a down-regulation in DK-MG cells. In view of the essential role of mTOR in cellular metabolism, including protein and phospholipid synthesis [45], it can be expected that the plasma membrane properties will be differently affected by MK-2206 in the two tested GBM cell lines. To prove this assumption, we examined the impact of MK-2206 and PI-103 on the plasma membrane folding and area by means of the contra-rotating field (CRF) technique [30]. Using this technique, we measured the area-specific plasma membrane capacitance *C*_m_ [μF/cm^2^] and the whole-cell capacitance *C*_C_ [pF]. *C*_m_ reflects the morphological complexity of the cell surface, such as membrane folds, protrusions and microvilli [44], whereas the parameter *C*_C_ accounts for the total electrically accessible cell membrane, including both smooth and folded membrane regions. The results of electrorotation experiments are shown in the Additional file 4: Figures S9, S10 and summarized in Additional file 2: Table S4.

As seen in Additional file 2: Table S4, the *C*_m_ value of control DK-MG cells (~ 2.5 μF/cm^2^) is much lower than that of SNB19 cells (~ 3.9 μF/cm^2^), which can be explained by a lower degree of membrane folding in DK-MG cells [44]. Treatment with the Akt inhibitor MK-2206 for 3 h significantly reduced both *C*_m_ and *C*_C_ of DK-MG cells by ~ 25% (2.5 → 1.86 μF/cm^2^) and ~ 33% (19.5 → 13.2 pF), respectively, indicating a marked decline of the plasma membrane area. In contrast and as expected, in SNB19 cells, MK-2206 reduced the *C*_m_ and *C*_C_ by ~ 15% (3.9 → 3.3 μF/cm^2^) and 25% (30.3 → 23.0 pF), respectively, to a lesser extent than in DK-MG cells.

Interestingly, PI-103 also reduced both *C*_m_ and *C*_C_ of DK-MG, but not as strong as MK-2206. Combined MK-2206 and PI-103 treatment caused the strongest observed reduction of *C*_m_ (− 30%) and *C*_C_ (− 40%) in DK-MG cells compared to controls, while no additional reduction of *C*_m_ was observed in SNB19 cells treated with both drugs. In the light of the recent finding that the plasma membrane turnover actively contributes to cell migration [46], the drug-induced reduction of the membrane surface area (probed by *C*_C_) in both cell lines (Additional file 2: Table S4), may at least partly be responsible for the observed drug-mediated inhibition of cell migration revealed by wound-healing assay (Fig. 1c, d).

## Discussion

Protein kinase B or Akt is the primary downstream mediator of PI3K signaling. It influences numerous cellular processes, including survival, growth, proliferation, angiogenesis, metabolism and migration [47]. The Akt family of kinases includes three members, Akt1, Akt2 and Akt3. Several studies have investigated the specific roles of individual Akt family members in cell migration and invasion [48, 49]. Interestingly, Akt1 has been found to function as an inhibitor of migration and invasion in breast [48] and ovarian [49] cancer cells, whereas inhibition of Akt2 has no effect on cell motility [48].

In the present study, we tested the effect of targeting Akt using the pan-Akt inhibitor MK-2206, which inhibits all three isoforms of Akt, with the intention of impeding the migration and radiation resistance of two GBM cell lines differing in *p53* and *PTEN* status [44], and therefore exhibiting different background expression of PI3K pathway proteins, including Akt. In addition, we examined whether MK-2206 can enhance the radiosensitizing effect of the dual PI3K/mTOR inhibitor PI-103. We found that MK-2206 strongly inhibited the expression of p-Akt in both cell lines (Fig. 5). However, contrary to expectations and despite depletion of p-Akt, MK-2206 did not radiosensitize both tumor cell lines but instead *increased* the radiation resistance of SNB19 cells (Fig. 2b, curve 3). Moreover, MK-2206 only moderately impeded SNB19 cell motility, but strongly affected the migration of DK-MG cells (Fig. 1). These results may be due to the following reasons: (*i*) MK-2206 did not induce cell cycle arrest (Fig. 3); (*ii*) neither did it induce excessive autophagy or apoptosis (Additional file 4: Figures S2, S3, Fig. 4); (*iii*) MK-2206 caused an aberrant activation of mTOR and its downstreams in SNB19 cells (Fig. 5); (*iv*) MK-2206 had no effect on the extent of induced and residual DNA damage in irradiated cells (Fig. 6). Furthermore, it did not enhance the radiosensitizing effect of PI-103 (Fig. 2).

The lack of a radiosensitizing effect of MK-2206 on GBM cell lines found in our study differs from the findings of Chautard et al. (2010) who showed a moderate increase of radiation sensitivity of two glioma cell lines treated with the Akt inhibitor IV (B2311) [21]. Likewise, our findings are different from those of Li et al. (2009) and Holler et al. (2016) who showed, respectively, an increased cell killing in colony-forming test of U87-MG and H460 cells treated with MK-2206 [20, 22]. These discrepancies might reside either in the particular Akt inhibitor, treatment protocol or the cell line used. Indeed, a differential response of tumor cells to full inhibition of Akt was observed and discussed elsewhere [22, 50]. For instance, in the study of Holler et al. (2016) in addition to a H460 cell line *responsive* to Akt inhibitor, there was a *non-responsive* A549 cell line which did not show any increased radiation sensitivity after Akt targeting [22].

**Fig. 8:**
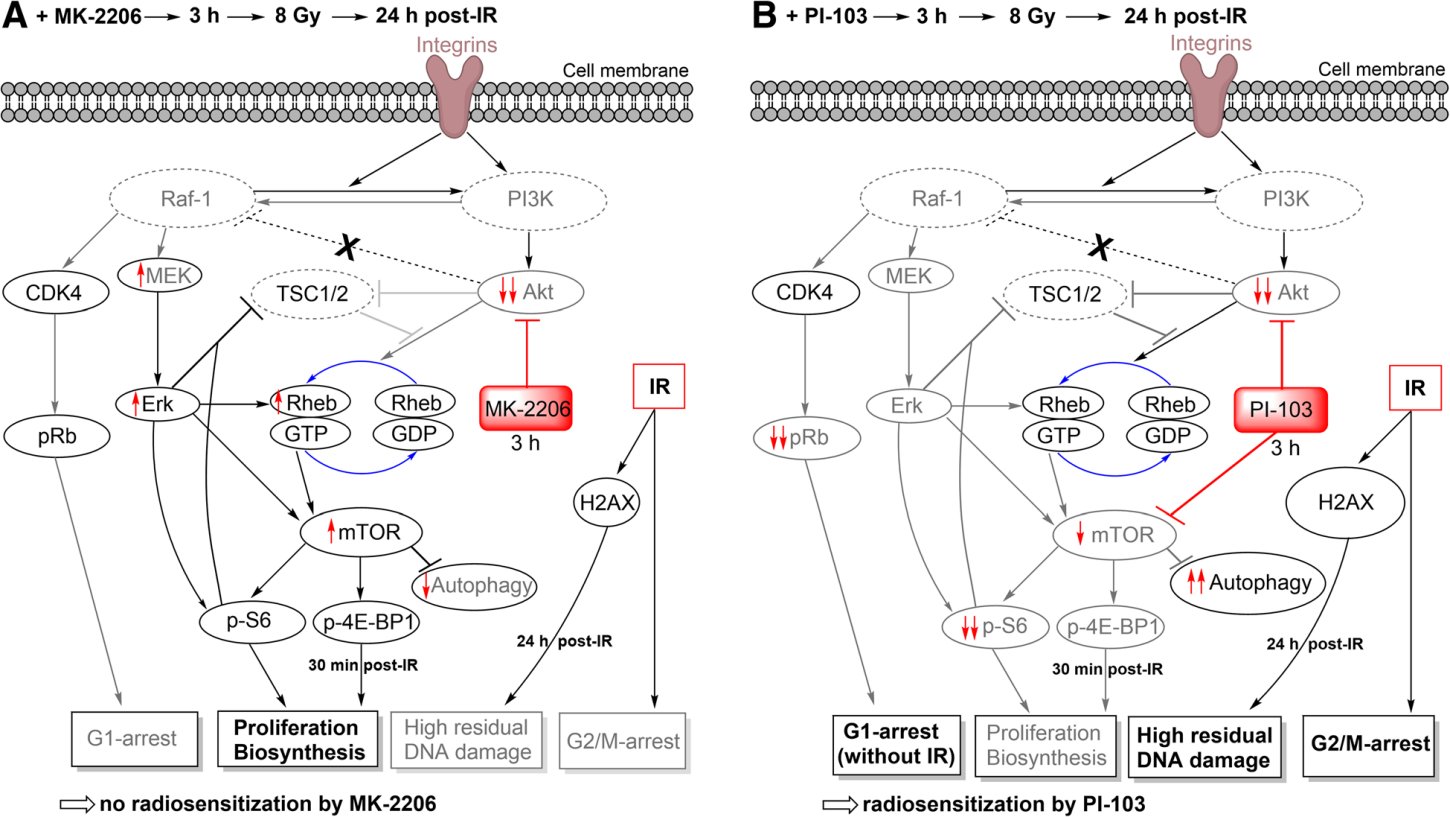
Schematic outline of possible signaling pathways responsible for different radiation responses of SNB19 cells pre-treated either by MK-2206 (**a**) or PI-103 (**b**). Despite strong depletion of p-Akt in MK-2206-treated cells, there was a moderate increase of its downstream p-mTOR (**a**). In contrast, p-mTOR was decreased in PI-103-treated cells (**b**) and consequently, its downstream effectors p-4E-BP1 and p-S6 were decreased as well (**b**). In sharp contrast, in MK-2206-treated cells the levels of p-4E-BP1 and p-S6 remain almost the same as in non-treated cells (**A**). In addition, inhibiting Akt by MK-2206 moderately stimulates the activity of MEK1/2 and Erk1/2 (**A**) whereas the effect was not seen after treatment with PI-103 (**b**). The proposed pathways are derived from the findings shown in Figs. 1, 2, 3, 4, 5, 6, 7 and Additional file 4: Figsure S1-S9, and also from previously published data [37]. Non-detected proteins are marked with dashed lines. Increased and decreased protein expression levels compared to corresponding control cells, are denoted by the symbols ↑ and ↓, respectively, as detected by Western blot analysis. (For details, *see* the Discussion section)

Based on our findings we suggest a simplified scheme presented in Fig. 8. Parts A and B of Fig. 8 illustrate the effects of MK-2206 (A) and PI-103 (B) on the radiation sensitivity of SNB19 cells. One of the possible reasons for the inability of MK-2206 to increase the radiation sensitivity of both cell lines, or in case of SNB19 cells to cause radioresistance, might be the activation of negative feedback loops leading to unimpeded or even increased mTOR function in MK-2206-treated cells (Fig. 5, line: 8 Gy, 30 min). As is known, Akt phosphorylates and thereby inhibits the heterodimer TSC1/TSC2, which in turn inhibits activation of the small GTPase Rheb, thus promoting mTORC1 activation [41]. Therefore, mTORC1 cannot be activated in drug-treated cells lacking p-Akt. As evident from Fig. 5, the expression of p-Akt was similarly depleted in cell samples treated shortly with either MK-2206 or PI-103. However, the expression of its downstream, i.e. mTOR was decreased only in PI-103-treated but not in MK-2206-treated SNB19 cells. Moreover, in MK-2206-treated SNB19 cells the expression of p-mTOR was even increased compared to PI-103-treated cells. The latter finding can be explained by the moderately increased expression of Rheb in SNB19 cells treated with MK-2206 (Additional file 4: Figure S6). Apparently, the activation of mTOR measured 30 min post-IR resulted in increased protein synthesis and subsequently enhanced colony-forming ability by (*a*) phosphorylating and *inactivating* the translational inhibitor 4E-BP1 and (*b*) by phosphorylating and *activating* S6 kinase in MK-2206-treated SNB19 cells. This in turn likely enhanced the radioresistance of SNB19 cells treated with the Akt inhibitor MK-2206. The same explanation applies to the moderate effect of MK-2206 on the migration of SNB19 cells reported here. Another reason for the differential effect of MK-2206 on the migration of DK-MG and SNB19 cells might be a different ratio of three Akt isoforms in these cell lines, which were not studied here separately. Subsequently, this may suggest the existence of isoform-specific effects on the molecular cascades that lead to the rearrangement of actin cytoskeleton, lamellipodium extension, and cell spreading, and MMPs (matrix-metalloproteinase) production [51]. Future works in this direction on an extended cell sample should bring more clarity in this matter.

Besides inhibition by Akt, the TSC1/2 complex, a negative regulator of mTORC1, can also be inhibited by the MAPK pathway [52]. Phosphorylation of TSC2 by Erk promotes dissociation of the tuberous sclerosis complex and attenuates TSC2-mediated inhibition of mTOR in cells. Therefore, a compensatory activation of the MAPK pathway (Additional file 4: Figure S5) in response to individual (here MK-2206) inhibitors [53] of the PI3K pathway can be another plausible explanation for the increased radiation resistance of SNB19 cells treated with the Akt inhibitor (Fig. 2b). It is known that both the PI3K and the MAPK pathway include critical factors for enhanced proliferation and survival of tumor cells [37] and constitutive activation of both pathways is correlated with a limited response to radiotherapy. These linear cascades rarely act as independent parallel pathways; rather they influence each other at different points and phases of signal propagation [54]. Because the MAPK pathway is responsible for cell survival and proliferation, we hypothesize that feedback activation of MEK/Erk proteins counteracts the cell cytotoxicity of Akt inhibition. Indeed, 30 min post-IR we observed an *increase* of p-MEK and p-Erk1/2 expressions, at least moderately, in MK-2206-treated samples (Additional file 4: Figure S5), which reflects the MAPK pathway *activation* following an Akt *inhibition*. An activation of Erk1/2 lead to the activation of Rheb and subsequently to the activation of mTORC1. Another reason for the inability of MK-2206 to increase the radiation sensitivity of both cell lines might be an activation of cytoprotective autophagy. As shown previously by Fan et al. (2010), PI-103 induces autophagy as a survival pathway in glioma cells [33]. Indeed, as seen in Additional file 4: Figure S3, PI-103, either alone or in combination with MK-2206, induced autophagy measured by the induction of LC3B-II or depletion of p62 proteins. However, the extent of (cytoprotective) autophagy induced by PI-103 was much higher compared to that induced by MK-2206 alone, especially in SNB19 cells. Thus, cytoprotective autophagy induced by MK-2206 alone can be ruled out as a reason for the increased radiation resistance of MK-2206-treated cells.

Further we found no differences in the induction and repair of DNA damage (Fig. 6) and the expression of DNA repair proteins (Fig. 7 and Additional file 4: Figure S7) between non-treated samples and those treated with MK-2206, with the exception of increased residual DNA damage in PI-103-treated SNB19 cells. The effect of PI-103 is corroborated by our previously published data [24]. Therefore, the enhanced survival of irradiated cells treated with MK-2206 can be explained by the absence of increased DNA damage and sufficient DNA repair. In addition, MK-2206 did not cause any cell cycle arrest in both cell lines (Fig. 3) thus allowing the cells unhampered proliferation and survival after IR.

## Conclusions

Our study provides a proof-of-concept that inhibition of Akt by MK-2206 might be a promising therapeutic strategy for increasing radiation sensitivity of tumor cells. However, the aberrant activation of mTOR in response to Akt inhibition in *PTEN* mutated cell lines, may counteract or even prevent, radiosensitization. Therefore, the therapeutic window needs to be carefully defined, or a combination of Akt and mTORC1 inhibitors should be considered.

## Additional files

Additional file 1: Supplemental Materials. The primary and secondary antibodies used in this paper. (DOCX 57 kb)

Additional file 2: **Table S1.** Cell cycle-phase distribution in control and irradiated (2 or 8 Gy) SNB19 tumor cells. The cells were fixed either 30 min or 24 h after IR, permeabilized, stained with propidium iodide, and analyzed for their DNA content by flow cytometry. Data are presented as means (± SD) from at least three independent experiments. For detailed description, *see* legend to Fig. 3. **Table S2.** Detection of γH2AX as a measure of DNA damage in SNB19 cells by flow cytometry. Mean γH2AX values are normalized to the non-irradiated control (30 min post-irradiation). The data are means ±SE from at least three independent experiments. **Table S3.** Cloning efficiencies and radiosensitivity parameters^**a**^ of in vitro irradiated tumor cell lines untreated and pretreated with the MK-2206 and PI-103 either alone or in combination. ^**a**^Mean (± SE) from at least three independent experiments; ^**b**^CF2 is the colony-forming ability at 2 Gy; ^**c**^D_10_ is the radiation dose required to reduce colony-forming ability by 10%; ^**d**^The growth inhibition factor IF_10_ was calculated as (D_10_ control)/(D_10_ + inh.). **Table S4.** Impact of MK-2206, PI-103 either alone or in combination on the area-specific plasma membrane capacitance *C*_m_, the whole-cell capacitance *C*_C_^*^ and cell radius. *The data derived from the ROT experiments shown in the Additional file 4: Figure S10 represent the means ± SE of at least 60 cells. (DOCX 63 kb)

Additional file 3: **Movie S1.** Cell trajectory analysis of control and drug-treated DK-MG and SNB19 tumor cells. Cells were imaged every 10 min by time-lapse microscopy using a Nikon BioStation IM-Q (Nikon, Melville, NY). For single-cell tracking experiments, about 10^4^ cells in 2 ml CGM were plated into a Petri dish (diameter 35 mm) and cultivated overnight. In each experiment, time-lapse images were acquired over a 23-h period, using a × 10 phase contrast objective. The initial position of each cell was set to the origin (0,0). Trajectories of control cells of both cell lines are shown on the left and MK-2206-treated cells on the right side, respectively for a 5 h timeframe. MK-2206-treated cells were pretreated for 3 h with the drug before recording. The movies are played back at a rate of 10 frames per second (sped up 6000× real time). (AVI 5121 kb)

Additional file 4: **Figure S1**. Western blot analysis of cell-cycle regulatory protein expression in drug-treated DK-MG and SNB19 cells, normalized to β-actin intensity (loading control). Numbers denote protein/β-actin ratios (details in Fig. 5 legend). **Figure S2**. Western blot analysis of PARP- and cleaved PARP-expression in drug-treated DK-MG and SNB19 cells. Numbers denote protein/β-actin ratios. **Figure S3**. Western blot analysis of autophagy marker proteins LC3B and p62 in drug-treated DK-MG and SNB19 cells. Numbers denote protein/β-actin ratios (details in Fig. 5 legend). **Figure S4**. Western blot analysis of β-actin expression for proteins shown in Fig. 5 and Fig.7 (details in Figs. 5 and 7 legends). **Figure S5**. Western blot analysis of p-MEK1/2 and p-Erk1/2 in drug-treated DK-MG and SNB19 cells. Numbers denote protein/β-actin ratios (details in Fig. 5). **Figure S6**. Western blot analysis of Rheb in drug-treated DK-MG and SNB19 cells. Numbers denote protein/β-actin ratios (details in Fig. 5 legend). **Figure S7**. Western blot analysis of DNA-repair proteins in drug-treated DK-MG and SNB19 cells. Numbers denote protein/β-actin ratios (details in Fig. 5 legend). **Figure S8**. Western blot analysis of β-actin expression for proteins depicted in Fig. S7 (details in Fig. 7 legend). **Figure S9**. Cumulative plots of radius-normalized *f*_c_ values (*f*_c_·a) vs. external conductivity of DK-MG (A) and SNB19 (B) cells were obtained by contra-rotating-field (CRF) technique. Symbols represent mean *f*_c_·a (± SD) values from 20 cells measured at ~ 10, 25 and 40 μS/cm. Lines are best fits of Eq. 2 to the data. The steeper line slopes for DK-MG cells (A) imply smaller *C*_m_ values compared to SNB19 cells (B). For *C*_m_ and *C*_C_ values, calculated with Eq. 3, see Additional file 2: Table S4. **Figure S10**. Impact of drug-treatment on cell radius, *C*_m_, and *C*_C_ values of DK-MG (A) and SNB19 (B) cells. “*” denotes significant difference at *p* < 0.05. “n.s.” means “not significant”. (DOCX 753 kb)

## Abbreviations

Cdk: Cyclin dependent kinase
CGM: Complete growth medium
CRF: Contra-rotating fields
DSB: DNA double-strand breaks
GBM: Glioblastoma multiforme
IR: Ionizing radiation
mTOR: Mammalian target of rapamycin
PI: Propidium iodide
PTEN: Phosphatase and tensin homologue
ROT: Electrorotation
RTK: Receptor tyrosinkinase
