# Supplementary material for: Differential effects of the Akt inhibitor MK-2206 on migration and radiation sensitivity of glioblastoma cells
Source: BMC Cancer. 2019 Apr 3;19:299. doi: 10.1186/s12885-019-5517-4 (PMC6446411; doi:10.1186/s12885-019-5517-4)
Supplement: Supplementary file 1 — Supplemental Materials. The primary and secondary antibodies used in this paper. (DOCX 57 kb) [file 12885_2019_5517_MOESM1_ESM.docx]

# Additional File 1:

# Supplemental Materials

The primary antibodies used were: rabbit polyclonal anti-PTEN, mouse monoclonal anti-phospho-Akt (Ser473), rabbit monoclonal anti-phospho-mTOR (Ser2448), rabbit polyclonal anti-phospho-S6 (Ser240/244), mouse monoclonal anti-phospho-4E-BP1, rabbit monoclonal anti-phospho-MEK1/2 (Ser217/221), rabbit monoclonal anti-p44/42 MAPK (ERK1/2), rabbit monoclonal anti-Rad51, rabbit polyclonal anti-PARP, rabbit monoclonal anti-ATM (D2E2), rabbit polyclonal anti-Ku80, rabbit polyclonal anti-LC3B, rabbit monoclonal anti-SQSTM1/p62 (D5E2), rabbit monoclonal anti-Rheb (E1G1R) (all from Cell Signaling, Danvers, MA), mouse monoclonal anti-Rad50 (13B3/2C6) (Abcam, Cambridge, UK), mouse monoclonal anti-Ku70 Ab-4 (Clone N3H10) (Thermo Fisher Scientific, Schwerte, Germany) mouse monoclonal anti-β-actin (Sigma, Deisenhofen, Germany) and mouse monoclonal anti-phospho-histone H2AX (Ser139) (Millipore, Schwalbach, Germany). Secondary species-specific antibodies for western blot were labelled with horseradish-peroxidase (DAKO, Hamburg, Germany).
