# Supplementary material for: Differential effects of the Akt inhibitor MK-2206 on migration and radiation sensitivity of glioblastoma cells
Source: BMC Cancer. 2019 Apr 3;19:299. doi: 10.1186/s12885-019-5517-4 (PMC6446411; doi:10.1186/s12885-019-5517-4)
Supplement: Supplementary file 2 — Table S1. Cell cycle-phase distribution in control and irradiated (2 or 8 Gy) SNB19 tumor cells. The cells were fixed either 30 min or 24 h after IR, permeabilized, stained with propidium iodide, and analyzed for their DNA content by flow cytometry. Data are presented as means (± SD) from at least three independent experiments. For detailed description, see legend to Fig. 3. Table S2. Detection of γH2AX as a measure of DNA damage in SNB19 cells by flow cytometry. Mean γH2AX values are normalized to the non-irradiated control (30 min post-irradiation). The data are means ±SE from at least three independent experiments. Table S3. Cloning efficiencies and radiosensitivity parametersa of in vitro irradiated tumor cell lines untreated and pretreated with the MK-2206 and PI-103 either alone or in combination. aMean (± SE) from at least three independent experiments; bCF2 is the colony-forming ability at 2 Gy; cD10 is the radiation dose required to reduce colony-forming ability by 10%; dThe growth inhibition factor IF10 was calculated as (D10 control)/(D10 + inh.). Table S4. Impact of MK-2206, PI-103 either alone or in combination on the area-specific plasma membrane capacitance Cm, the whole-cell capacitance CC* and cell radius. *The data derived from the ROT experiments shown in the Additional file 4: Figure S10 represent the means ± SE of at least 60 cells. (DOCX 63 kb) [file 12885_2019_5517_MOESM2_ESM.docx]

**Additional File 2:**

**Supplemental (S) Tables:**

**Table S1:**

| **Cell line** | **Treatment modality** | | | **G1 (%)** | **S (%)** | **G2/M (%)** | **G2/G1** |
| --- | --- | --- | --- | --- | --- | --- | --- |
| **SNB19** | 30 min | 0 Gy |  | 26.6±1.3 | 49.5±4.5 | 23.9±5.2 | 0.9 |
|  |  | 2 Gy |  | 27.0±1.4 | 50.6±5.0 | 22.4±5.4 | 0.8 |
|  |  | 8 Gy |  | 27.8±1.7 | 49.9±4.9 | 22.3±4.7 | 0.8 |
|  | 24 h | 0 Gy |  | 43.3±1.7 | 33.9±3.3 | 22.8±1.6 | 0.5 |
|  |  | 2 Gy |  | 40.7±2.6 | 39.5±0.2 | 19.8±2.7 | 0.5 |
|  |  | **8 Gy** |  | **35.8±3.2** | **11.3±2.4** | **52.8±5.3** | **1.5** |

**Table S2:**

| **Irradiation dose** | **Mean γH2AX content** | |
| --- | --- | --- |
|  | **30 min post-IR** | **24 h post-IR** |
| 0 Gy | 1.0 | 0.80±0.02 |
| 2 Gy | 1.50±0.04 | 0.90±0.06 |
| **8 Gy** | **2.90±0.11** | **1.3±0.09** |

## Table S3:

| **Cell line** | **Plating efficiency** | **CF2 ^b^** | **D_10_ (Gy)^c^** | **IF_10_^d^**  **(D_10_ control)/**  **(D_10_+inh.)** |
| --- | --- | --- | --- | --- |
| **DK-MG** – contr. | 0.10±0.01 | 0.58±0.07 | 7.2±1.0 | 1.0 |
| PI-103 | 0.07±0.01 | 0.43±0.08 | 5.6±1.0 | 1.3±0.1 |
| MK-2206 | 0.12±0.02 | 0.57±0.04 | 6.5±0.5 | 1.1±0.2 |
| both | 0.06±0.01 | 0.44±0.10 | 4.6±0.6 | 1.6±0.6 |
| **SNB19** – contr. | 0.11±0.02 | 0.75±0.04 | 7.3±0.3 | 1.0 |
| PI-103 | 0.09±0.04 | 0.45±0.05 | 5.2±0.6 | 1.4±0.1 |
| MK-2206 | 0.11±0.03 | 0.8±0.04 | 9.9±1.0 | 0.7±0.2 |
| both | 0.08±0.03 | 0.50±0.05 | 5.4±0.5 | 1.4±0.2 |

## Table S4:

|  |  | ***C*_m_, µF/cm^2^** | ***C*_c_, pF** | **Cell radius, µm** |
| --- | --- | --- | --- | --- |
| **DK-MG** | control | 2.49 ±0.17 | 19.58±2.13 | 7.91±0.16 |
|  | MK-2206 | 1.86 ± 0.07 | 13.22±0.85 | 7.52±0.10 |
|  | PI-103 | 2.21 ± 0.13 | 14.92 ± 1.28 | 7.33 ± 0.10 |
|  | PI-103+MK-2206 | 1.74 ± 0.09 | 11.97 ± 1.01 | 7.40 ± 0.12 |
| **SNB19** | control | 3.89 ± 0.36 | 30.28 ± 3.88 | 7.87 ± 0.14 |
|  | MK-2206 | 3.34 ± 0.16 | 22.98 ± 1.47 | 7.40 ± 0.06 |
|  | PI-103 | 3.12 ± 0.18 | 23.37 ± 2.01 | 7.72 ± 0.11 |
|  | PI-103+MK-2206 | 3.52 ± 0.28 | 23.12 ± 2.54 | 7.23 ± 0.11 |
