# Supplementary figures and images for: Differential effects of the Akt inhibitor MK-2206 on migration and radiation sensitivity of glioblastoma cells
Source: BMC Cancer. 2019 Apr 3;19:299. doi: 10.1186/s12885-019-5517-4 (PMC6446411; doi:10.1186/s12885-019-5517-4)

**Additional File 3:**

**Supplemental (S) Figures (Fig):**

**Fig S1:**

| **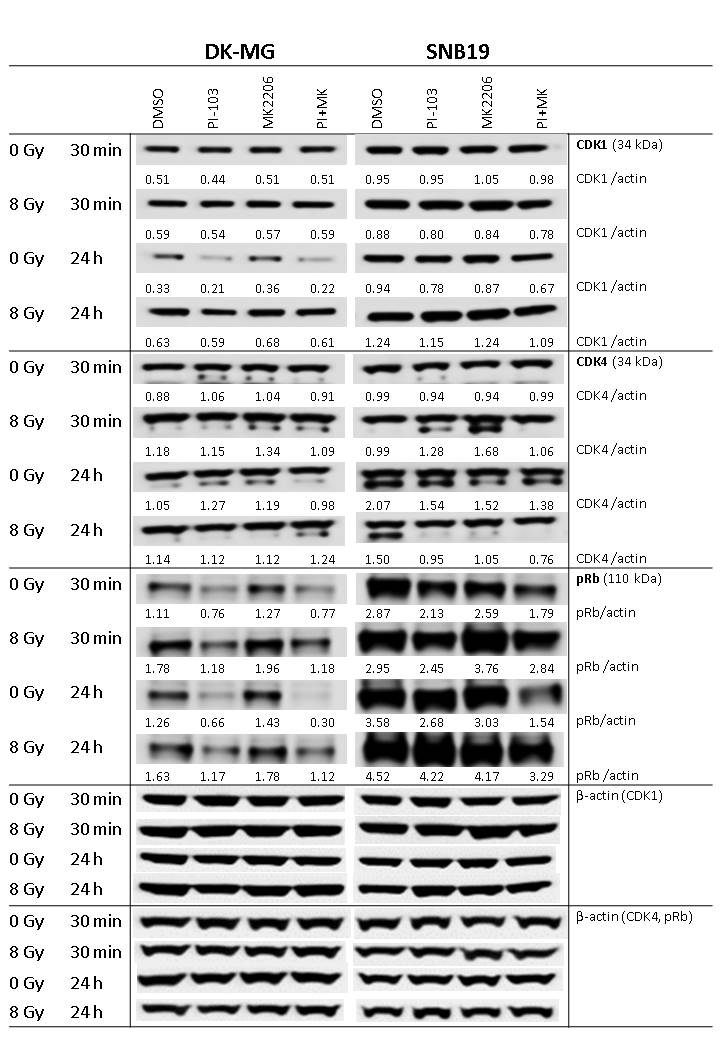** |
| --- |
| **Fig. S2:**  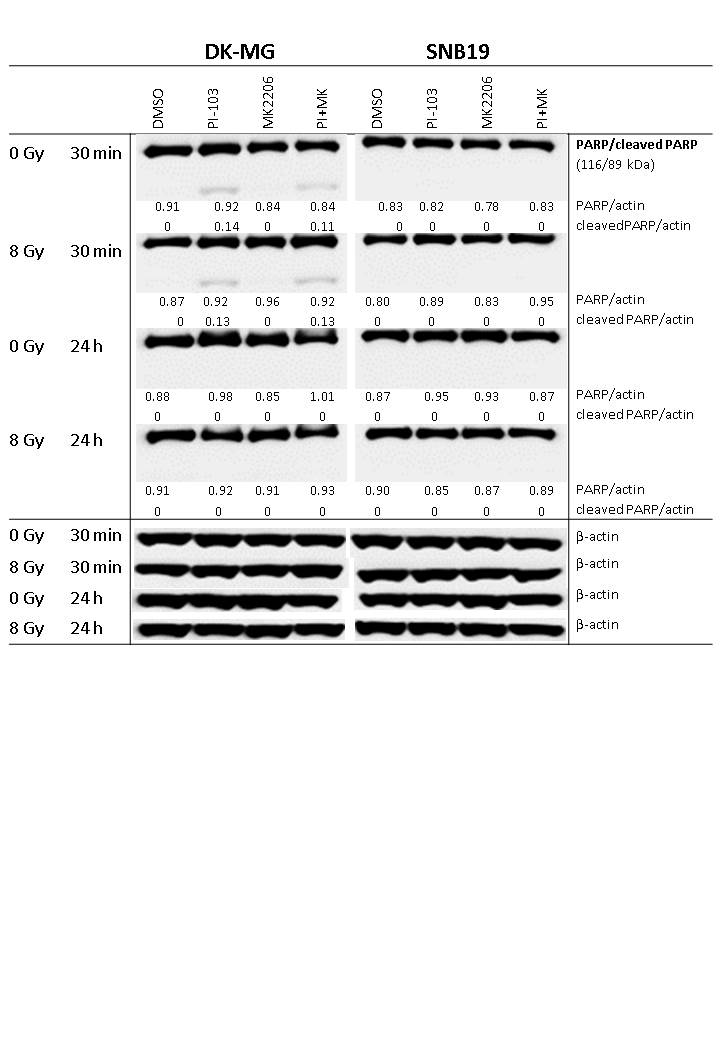 |
| **Fig. S3:**  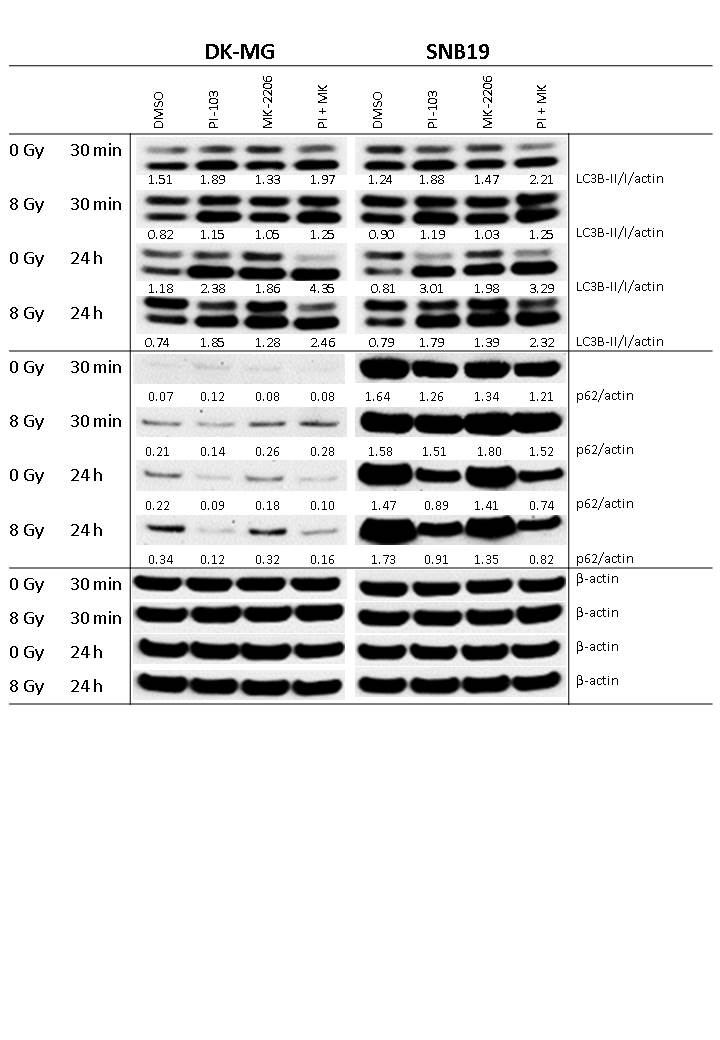 |

| **Fig. S4:**  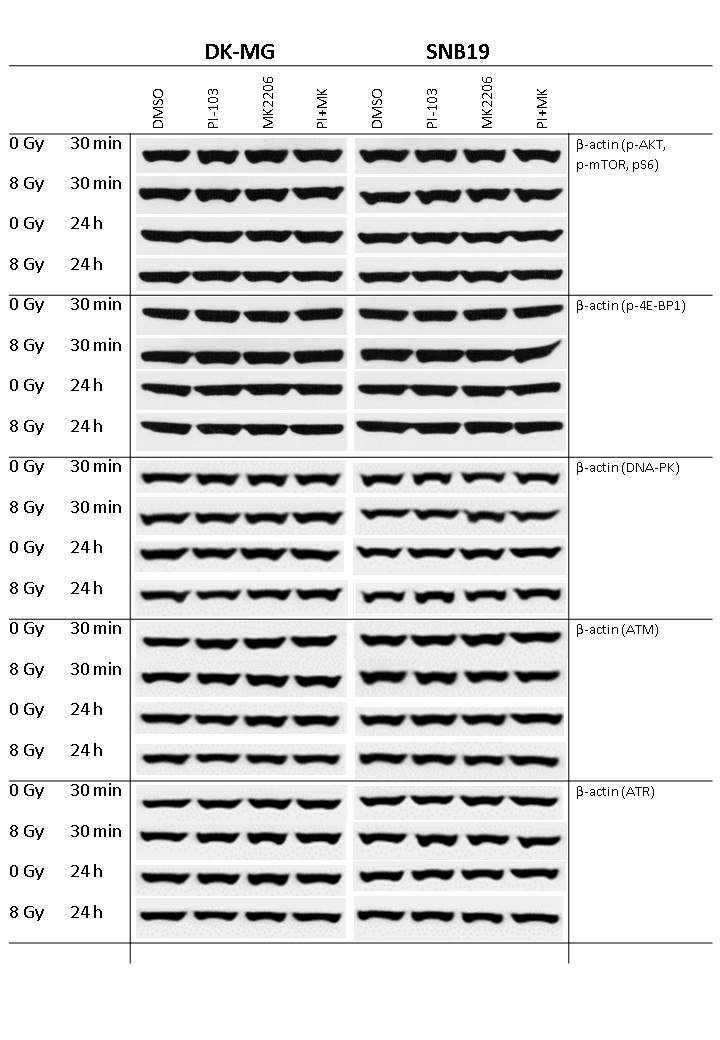 | |
| --- | --- |
| 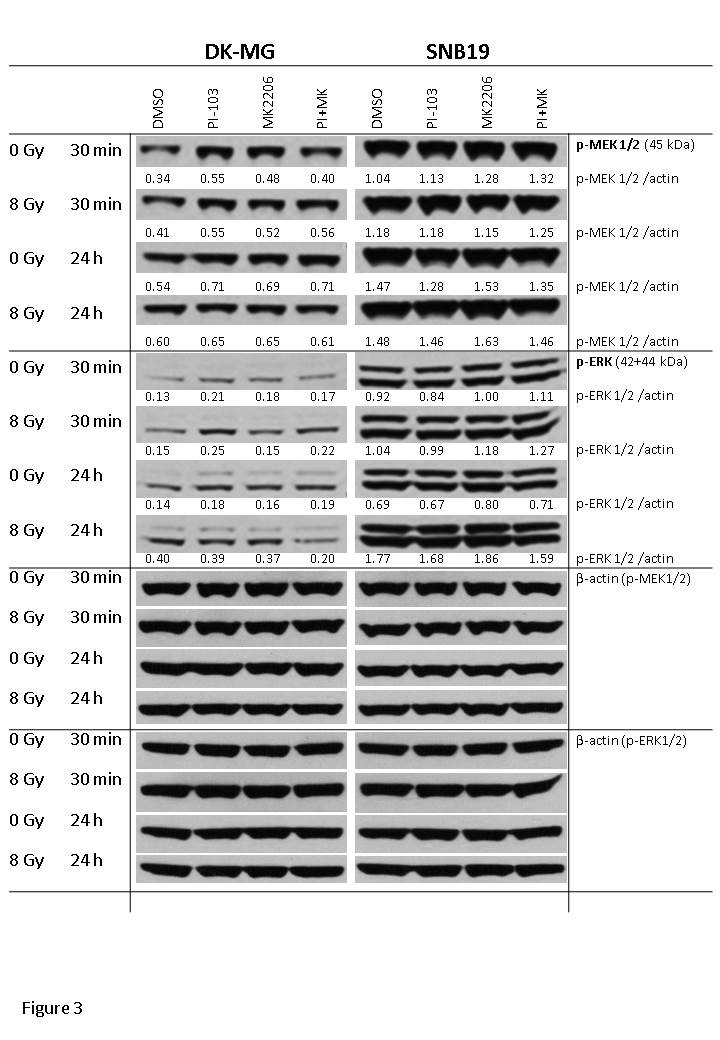**Fig. S5:** |

| 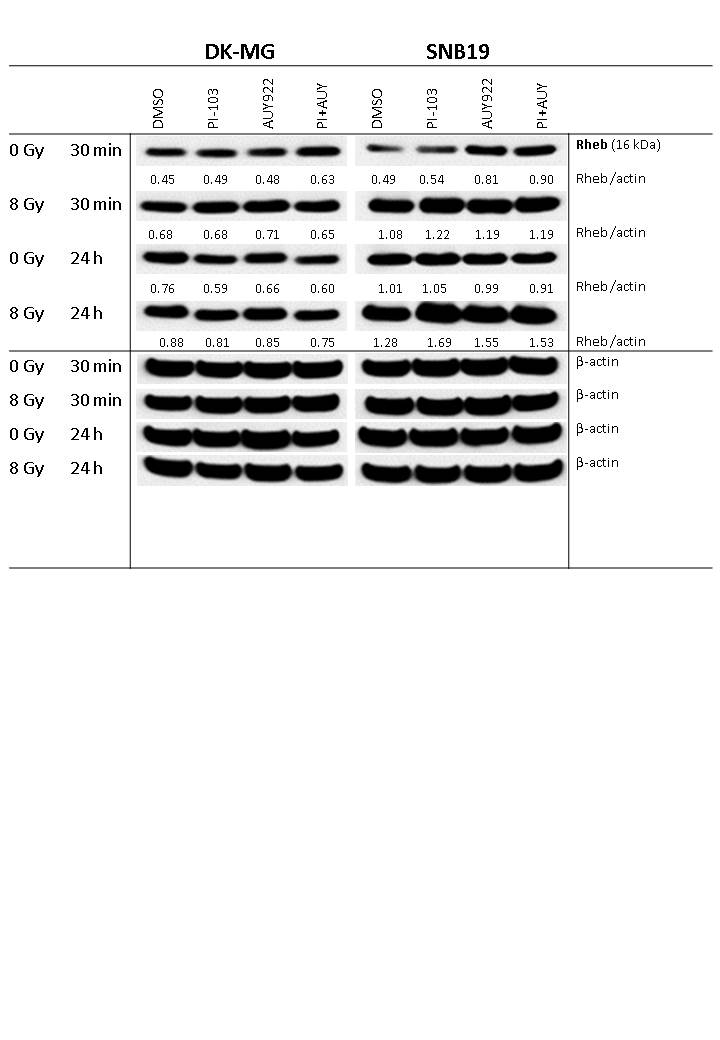**Fig. S6:** |
| --- |

| 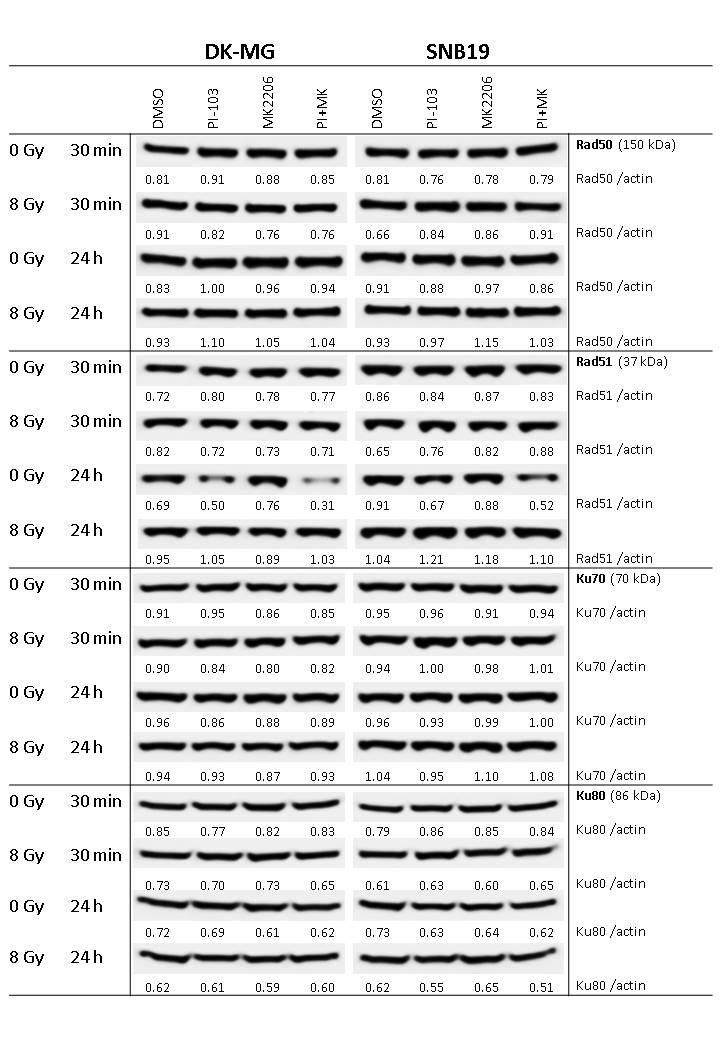**Fig. S7:** |
| --- |

| **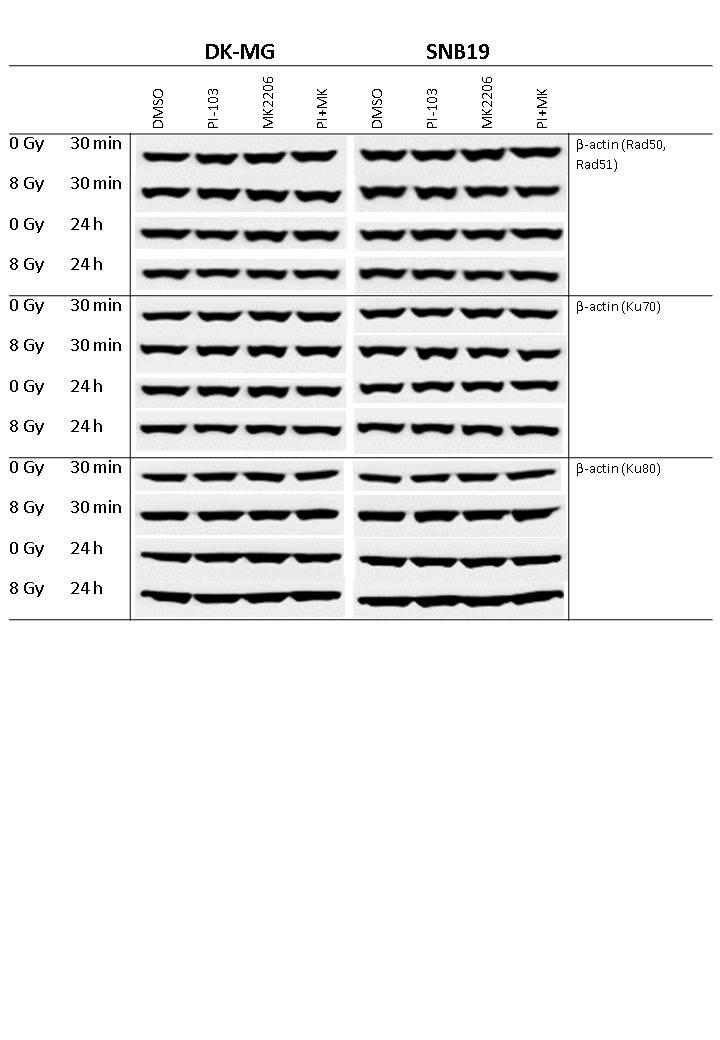Fig. S8:** | | |
| --- | --- | --- |
|  | | |
| **Fig. S9:** | | |
|  | | |
| 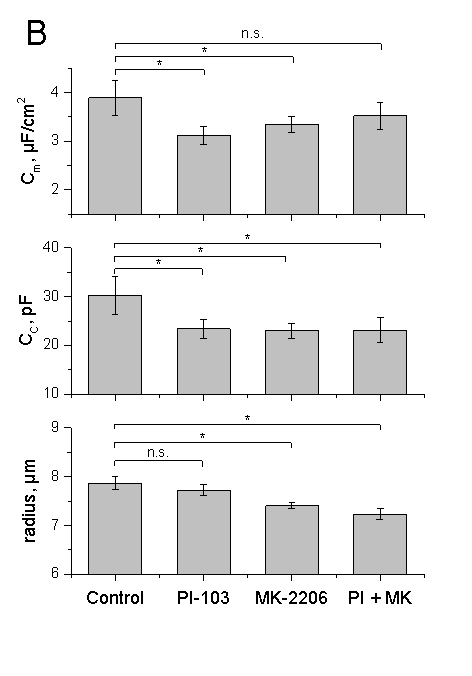**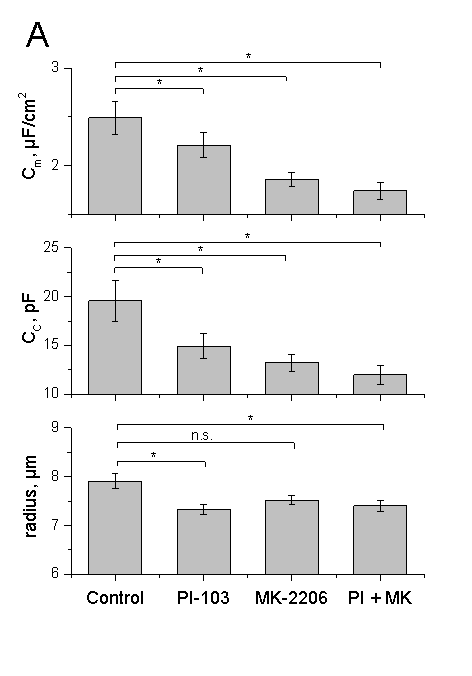Fig. S10:** |  |
|  | |

## 

Supplement: Supplementary file 4 — Figure S1. Western blot analysis of cell-cycle regulatory protein expression in drug-treated DK-MG and SNB19 cells, normalized to β-actin intensity (loading control). Numbers denote protein/β-actin ratios (details in Fig. 5 legend). Figure S2. Western blot analysis of PARP- and cleaved PARP-expression in drug-treated DK-MG and SNB19 cells. Numbers denote protein/β-actin ratios. Figure S3. Western blot analysis of autophagy marker proteins LC3B and p62 in drug-treated DK-MG and SNB19 cells. Numbers denote protein/β-actin ratios (details in Fig. 5 legend). Figure S4. Western blot analysis of β-actin expression for proteins shown in Fig. 5 and Fig.7 (details in Figs. 5 and 7 legends). Figure S5. Western blot analysis of p-MEK1/2 and p-Erk1/2 in drug-treated DK-MG and SNB19 cells. Numbers denote protein/β-actin ratios (details in Fig. 5). Figure S6. Western blot analysis of Rheb in drug-treated DK-MG and SNB19 cells. Numbers denote protein/β-actin ratios (details in Fig. 5 legend). Figure S7. Western blot analysis of DNA-repair proteins in drug-treated DK-MG and SNB19 cells. Numbers denote protein/β-actin ratios (details in Fig. 5 legend). Figure S8. Western blot analysis of β-actin expression for proteins depicted in Fig. S7 (details in Fig. 7 legend). Figure S9. Cumulative plots of radius-normalized fc values (fc·a) vs. external conductivity of DK-MG (A) and SNB19 (B) cells were obtained by contra-rotating-field (CRF) technique. Symbols represent mean fc·a (± SD) values from 20 cells measured at ~ 10, 25 and 40 μS/cm. Lines are best fits of Eq. 2 to the data. The steeper line slopes for DK-MG cells (A) imply smaller Cm values compared to SNB19 cells (B). For Cm and CC values, calculated with Eq. 3, see Additional file 2: Table S4. Figure S10. Impact of drug-treatment on cell radius, Cm, and CC values of DK-MG (A) and SNB19 (B) cells. “*” denotes significant difference at p < 0.05. “n.s.” means “not significant”. (DOCX 753 kb) [file 12885_2019_5517_MOESM4_ESM.docx]
